# Decomposing sources of value for electricity and negative emissions technologies in net-zero power systems

**DOI:** 10.1016/j.isci.2025.114187

**Published:** 2025-12-09

**Authors:** Daniel C. Steinberg, Daniel P. Cherney, Bryan K. Mignone, Matthew Mowers, Brian Sergi

**Affiliations:** 1 National Laboratory of the Rockies, Strategic Energy Analysis Center, Golden, CO, USA; 2 ExxonMobil Technology & Engineering Company, Annandale, NJ, USA; 3 Matthew Mowers, LLC, Independent Consultant, New York, NY, USA

**Keywords:** environmental science, engineering, energy sustainability

## Abstract

Deep decarbonization of the US power system would require rapid deployment of variable renewable energy (VRE) resources, which are projected to provide a substantial share of electricity generation at the time of net-zero emissions. However, the exact share of generation met by VRE and the roles of other technologies in supplying key electricity services—energy and firm capacity—remain uncertain. This study employs a detailed model of the US power sector to decompose the provision and value of electricity services, including negative emissions, by technology across a range of deep decarbonization scenarios. Results indicate that while technology deployment and the share of services provided by each technology vary significantly depending on future technological and market conditions, the value composition and future roles of individual technologies remain consistent. These findings offer guidance for research and development priorities and provide insights to inform electricity policy and planning.

## Highlights

• VRE, nuclear, and fossil generation with CCS are mostly deployed for energy
• Existing unabated fossil and new combustion turbines largely provide firm capacity
• Storage and combined cycle plants provide energy and firm capacity
• CDR offsets emissions that enable peaking energy contributions from fossil sources

## Graphical abstract

**Figure:**
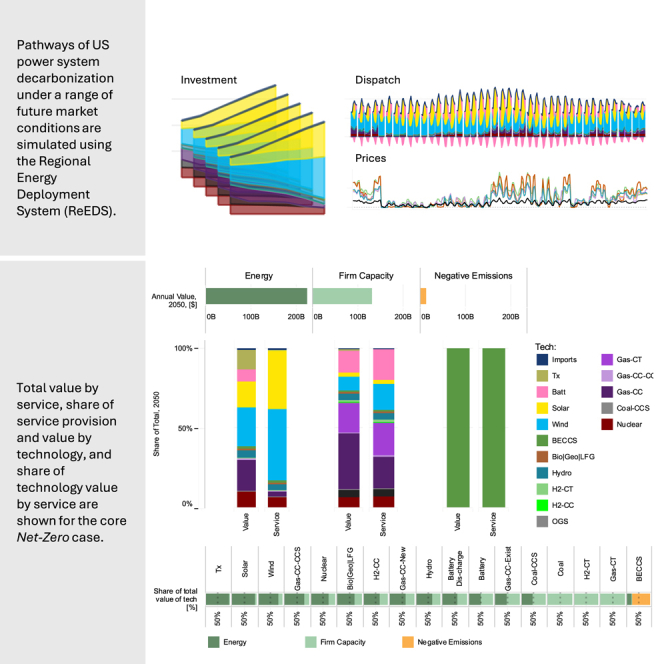

## Introduction

Scenarios consistent with climate stabilization near 1.5°C or 2°C typically attain net-zero carbon dioxide (CO_2_) emissions between 2050 and 2070.^1^ A key element of such scenarios is the electrification of end-use services combined with the near elimination of emissions from the power sector.^2^^,^^3^^,^^4^^,^^5^ This finding has motivated a number of studies to explore the challenges, costs, and benefits of achieving a power system with low to negative carbon emissions.^3^^,^^6^^,^^7^^,^^8^^,^^9^^,^^10^^,^^11^^,^^12^^,^^13^^,^^14^^,^^15^^,^^16^ These studies demonstrate that multiple technology pathways could be consistent with deep emissions reductions,^2^^,^^4^^,^^9^^,^^10^^,^^15^^,^^17^^,^^18^^,^^19^^,^^20^ albeit with important cost implications.^7^^,^^8^^,^^9^^,^^14^

At a high level, decarbonization pathways in the electricity sector are typically characterized by the rapid deployment of variable renewable energy (VRE) resources—wind and solar—such that, at the time of low, net-zero, or net-negative emissions, VRE constitutes a substantial share of total generation^2^^,^^4^^,^^9^^,^^15^^,^^18^^,^^19^^,^^20^^,^^21^^,^^22^^,^^23^^,^^24^^,^^25^ with recent studies^2^^,^^9^^,^^19^^,^^20^^,^^21^^,^^25^^,^^26^ indicating that VRE comprises the largest share. The remaining generation is composed of contributions from a suite of other technologies, including natural gas with or without carbon capture and storage (CCS), nuclear, hydroelectric, geothermal, biomass, and hydrogen generation. These non-VRE technologies generally account for most firm capacity used to meet resource adequacy requirements and related peaking energy needs.^9^^,^^10^^,^^14^^,^^23^ In addition, in many net-zero and net-negative scenarios, carbon dioxide removal (CDR) technologies such as biopower with carbon capture and storage (BECCS) or direct air capture with CO_2_ storage (DACCS) provide negative emissions to offset remaining emissions in the power system, thereby enabling firm capacity and a limited amount of generation from fossil sources.^8^^,^^9^^,^^19^^,^^20^

Despite many similarities in key aspects of electricity sector mitigation pathways, there remains uncertainty in the overall VRE and non-VRE shares of generation,^9^ the technology composition of the non-VRE share, and the future value and revenue streams of such technologies. Some of this uncertainty is related to uncertainty in future technology costs, fuel prices, load, and other economic and social drivers. However, the mix of technologies deployed also reflects differences in their potential roles in providing the suite of required grid services,^27^ particularly energy and firm capacity.

While prior studies have evaluated alternative decarbonization pathways under broader system constraints^2^^,^^4^^,^^8^^,^^9^^,^^10^^,^^15^^,^^17^^,^^18^^,^^21^^,^^22^ and examined the roles of individual technologies,^18^^,^^19^^,^^20^^,^^23^^,^^24^^,^^26^^,^^28^^,^^29^^,^^30^^,^^31^^,^^32^^,^^33^^,^^34^^,^^35^ these studies have focused on the physical provision of energy, the total capacity needed, and associated costs. Relatively few studies evaluate and disaggregate the value (or revenue) associated with these services,^20^^,^^28^^,^^36^ and those that do generally focus on a specific technology category. For example, Bistline and Young^20^ decompose the values of energy and capacity contributions of existing natural gas combined cycle (Gas-CC) technologies under decarbonized futures, and Mallapragada, Sepulveda, and Jenkins^28^ decompose the values of battery storage into avoided operational costs and avoided generation and transmission capacity investment. Mowers, Mignone, and Steinberg^36^ evaluate the value contributions of a broader range of generation technologies—specifically VRE, natural gas, and nuclear technologies—but do not evaluate other key generation (e.g., hydrogen), storage, or CDR technologies. Furthermore, they do not disaggregate the value of technologies into specific services.

In this study, using a spatially and temporally detailed national-scale planning model of the US power sector, the Regional Energy Deployment System (ReEDS) model, we quantify and decompose the physical provision of and value of key electricity services by technology across a range of zero to negative emissions scenarios. We focus on the two electricity services of greatest value—energy and firm capacity—as well as the provision of negative emissions, and evaluate all major generation, storage, transmission, and CDR technologies. Decomposing both the physical provision and value of electricity services by technology informs our understanding of the roles that different technologies could play in such scenarios.

A key strength of our approach is that we systemically consider a suite of scenarios that vary market and policy conditions that would be more or less favorable to VRE and several non-VRE technologies, including natural gas with CCS, hydrogen, and CDR. This allows us to evaluate conditions under which those technologies might deploy and how they derive revenue under alternative future carbon constrained electricity systems. We are not aware of a consistent comparison of these options in a single study focusing on conditions for their deployment and the mechanisms by which they could derive revenue in deep decarbonization scenarios.

Our results suggest that while the overall deployment of specific technologies will depend on future technology, policy, and market conditions, their roles—suggested by their value compositions—are largely robust to such conditions. In deep mitigation scenarios, VRE technologies, which make up the largest share of generation across all cases, derive most of their value from energy provision. Among non-VRE technologies, fossil generation with CCS and nuclear, when deployed, are largely deployed to provide energy and thereby predominantly compete with VRE. Unabated fossil and hydrogen primarily provide firm capacity and peaking energy. CDR technologies, including both biopower with CCS and DAC, derive value from providing negative emissions that enable contributions from fossil generation, both with and without CCS. Such insights can inform policy design, energy system planning decisions, and technology research and development priorities.

## Results

### Electricity service provision

ReEDS represents requirements for three types of key electricity services—energy, firm capacity, and operating reserves. While all three services are necessary to achieve reliable operation, the total value of operating reserves relative to energy and firm capacity is small, so we focus on energy and firm capacity. This section presents the physical provision of these services, whereas the next section discusses the value of these services and how technology value differs from technology deployment and service provision.

**Figure 1:**
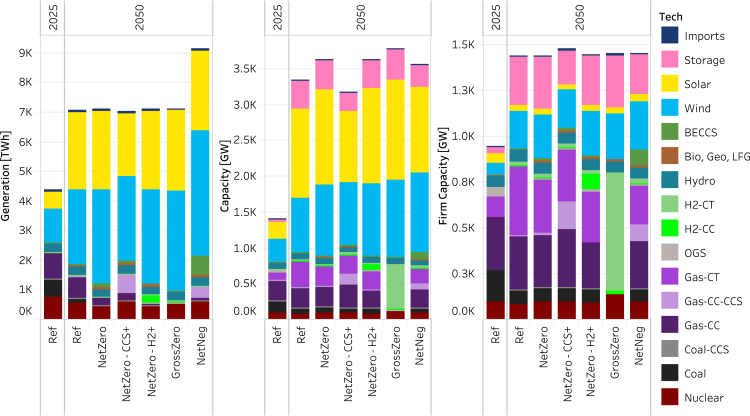
Generation, capacity, and firm capacity in the core cases in 2025 and 2050 2025 results are shown only for the reference case, as results are identical across scenarios in 2025. Imports are net imports from Canada. Storge and transmission losses are not shown. The results for sensitivity cases are shown in Figure S7. The generation and capacity by year for each core case and sensitivity case are shown in Figures S8 and S9, respectively. Dispatch for each core case is shown in Figures S10–S15.

Scenarios are defined in Table 1 and described in detail in the STAR Methods, scenarios section. Figure 1 shows the capacity, generation, and firm capacity mix across the core scenarios in 2025 and 2050. In all cases, total capacity, generation, and firm capacity grow significantly by 2050, largely driven by increases in electricity consumption. VRE makes up most of the growth in capacity and generation across all scenarios, comprising 70%–85% of total generation by 2050. This demonstrates that VRE technologies, supported by diurnal storage, are generally the most cost-effective options for meeting energy demand across a range of technology, market, and policy conditions.

**Table 1:** Scenario definitions

Scenario	CO_2_ Emissions Constraint	RE & Batt Cost & Performance	Gen-CCS & DACCS Cost & Performance	NG Price	H_2_ Price	Additional Assumptions
Reference (Ref)	No requirement	ATB23-Mid	Brown et al.-Mid^37^	AEO23-Ref	$2.30 per kg	–
NetZero	**Net-zero** by 2050	ATB23-Mid	Brown et al.-Mid	AEO23-Ref	$2.30 per kg	–
NetZero-H2+	**Net-zero** by 2050	ATB23-Mid	Brown et al.-Mid	**AEO23-LOG**	**$1.00 per kg**	–
NetZero-CCS+	**Net-zero** by 2050	**ATB23-Cons**	**Brown et al.-Adv**	**AEO23-HOG**	$2.30 per kg	–
GrossZero	**Gross-zero** by 2050	ATB23-Mid	**Unavailable in 2050**	AEO23-Ref	$2.30 per kg	**Minimum 1% capacity factor for thermal techs**
NetNeg	**Net-negative** 1 Gt by 2050	ATB23-Mid	Brown et al.-Mid	AEO23-Ref	$2.30 per kg	**Increased bioenergy supply**

Ref-EndogH2	No requirement	ATB23-Mid	Brown et al.-Mid	AEO23-Ref	**Endogenous**	–
NetZero-EndogH2	**Net-zero** by 2050	ATB23-Mid	Brown et al.-Mid	AEO23-Ref	**Endogenous**	–
GrossZero-EndogH2	**Gross-zero** by 2050	ATB23-Mid	**Unavailable in 2050**	AEO23-Ref	**Endogenous**	**Minimum 1% capacity factor for thermal techs**
Ref-EndogH2 & EconH2	No requirement	ATB23-Mid	Brown et al.-Mid	AEO23-Ref	**Endogenous**	**High ROE H**_**2**_**demand**
NetZero-EndogH2 & EconH2	**Net-zero** by 2050	ATB23-Mid	Brown et al.-Mid	AEO23-Ref	**Endogenous**	**High ROE H**_**2**_**demand**
GrossZero-EndogH2 & EconH2	**Gross-zero** by 2050	ATB23-Mid	**Unavailable in 2050**	AEO23-Ref	**Endogenous**	**High ROE H**_**2**_**demand; Minimum 1% capacity factor for thermal techs**
NetZero-HiDem	**Net-zero** by 2050	ATB23-Mid	Brown et al.-Mid	AEO23-Ref	$2.30 per kg	**High load**

The core scenarios are defined in the top six-rows; sensitivities to the core scenarios are defined in the rows 7-13. Bolded text indicates differences from the *Ref* scenario. ‘AEO23-Ref' refers to the 2023 Annual Energy Outlook Reference scenario. ‘AEO23-HOG' and ‘AEO23-LOG' refer to the High Oil and Gas Supply and Low Oil and Gas Supply scenarios, respectively.

However, the VRE share of total firm capacity is substantially smaller than its share of total generation and total capacity (Figure 4). While VRE accounts for most capacity, it provides just 16%–21% of total firm capacity. In contrast, while unabated natural gas capacity makes up only 13%–19% of total capacity and 11% or less of total generation across scenarios that allow fossil in 2050, it provides 32%–46% of total firm capacity. The sensitivity cases (Figures S6 and S7) generally exhibit firm capacity contributions from VRE and natural gas technologies within these ranges. However, in the *NetZero-EndogH2 & EconH2* case, despite a large increase in total load and generation, there is a negligible change in the total firm capacity requirement. This demonstrates that new loads, if flexible with sufficient duration potential (multi-day in the case of hydrogen), may have little impact on the firm capacity requirement. Furthermore, under this case, we observe a reduction in the firm capacity contribution from unabated gas, in particular from natural gas combustion turbine capacity, with an offsetting increase in the contribution from VRE and, to a lesser extent, storage. This effect is driven jointly by the increased deployment of VRE technologies to meet the increased energy requirement and the highly flexible load associated with hydrogen production, leading to increased VRE availability. A similar effect is demonstrated by the changes in firm capacity shares of H2-CT, VRE, and storage between the GrossZero and GrossZero-EndogH2 & EconH2 cases—the firm capacity share of H2-CT declines with a compensating increase from VRE and storage. Conversely, under the *NetZero-HighDemand* case, the increase in demand without any available flexibility, combined with the changes in the timing of the peaks, leads to an increase in supply-side resources with unabated natural gas technologies comprising 54% of total firm capacity.

While VRE makes up the majority share of generation, as shown in previous work, the marginal relative value of VRE declines as its share increases.^36^ As a result, other technologies provide substantive contributions to meeting energy demand. Of the non-VRE share of generation, incumbent nuclear and hydroelectric capacity consistently contribute between 10% and 15% of total generation across all scenarios, driven by their relatively low marginal operating costs and zero direct emissions.

**Figure 2:**
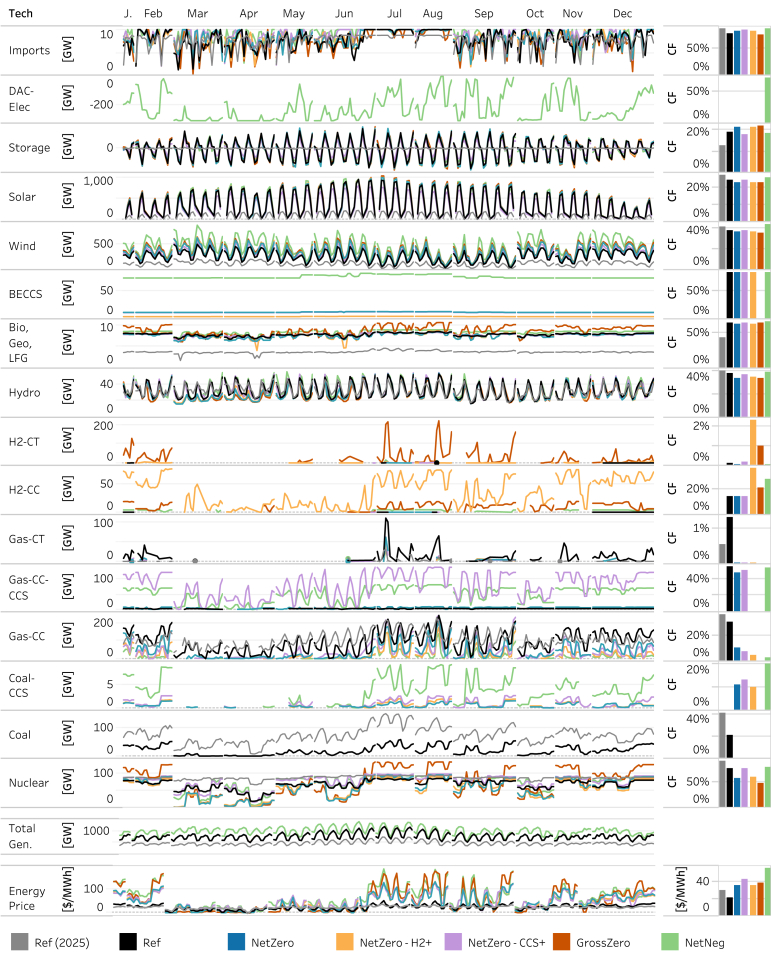
National aggregate technology dispatch and energy price profiles across scenarios in 2025 and 2050 2025 results are shown only for the reference case as results are identical across scenarios in 2025. Main panel: technology dispatch by time slice and scenario, and right-hand column: annual capacity factor (CF) by technology and scenario; bottom row: energy price by time slice and scenario, and generation-weighted average energy price by scenario. Imports are net imports from Canada. The CF for VRE technologies is calculated as the ratio of total annual energy delivered to the grid to the product of installed capacity and 8760 hours. The CF for storage is reported as the ratio of the total annual dispatch to the maximum potential dispatch unconstrained by state of charge—the product of capacity and 8760 hours. Figure S24 depicts average energy prices by year and scenario.

In addition, BECCS is deployed across all cap cases when allowed and operated near its maximum capacity factor (Figure 2), given its high capital cost and the value derived from providing negative emissions. Its role is most noticeable in the *NetNeg* case, given the need for substantial negative emissions in that case.

The composition of the remaining generation share—and the specific roles for natural gas CCS, unabated natural gas, and hydrogen—vary depending on assumptions about technology, fuel prices, and policy. In the *Ref* case, unabated natural gas combined cycle units provide most of the remaining generation—about 10% in 2050, which is down from 20% in 2025 (The 2023 historical annual generation share of natural gas was 43% (U.S. Energy Information Administration)). However, unabated fossil technologies are less competitive as an energy source in the cap cases due to the cost of associated carbon emissions. As a result, the share of unabated natural gas combined cycle generation falls to 3% or less across all cap cases; increased contributions arise from a mix of low, zero, and negative emission sources, including additional VRE, Gas-CC-CCS, H2-CC, and BECCS. Despite the reduction in unabated Gas-CC generation, the total capacity of Gas-CC remains relatively flat over time (Figures S8 and S9) across the *Ref* and most cap cases (except for *GrossZero,* which disallows fossil, and the *NetZero-HighDemand* sensitivity in which load is 60% higher in 2050). Gas-CT capacity expands substantially relative to 2025 in the same cases. In all scenarios that allow emitting generation, unabated natural gas remains cost-effective relative to other firm capacity options, such as hydrogen, for balancing the system at longer timescales.

Nonetheless, hydrogen plays a substantial role in two of the core cases: the *GrossZero* case and the *NetZero-H2+* case. In the *NetZero-H2+* scenario, lower hydrogen prices and higher natural gas prices favor the expansion of new H2-CC. H2-CC accounts for 4% of total generation in 2050, comparable to the share of Gas-CC in the *NetZero* case. The increased deployment of H2-CC capacity results in greater firm capacity contributions from H2-CC and lower firm capacity contributions from unabated natural gas. However, a significant amount of unabated natural gas capacity remains to provide firm capacity, as in the other cap cases in which emitting generation is allowed.

In the *GrossZero* case, all fossil generation is retired because it would otherwise be forced to run (given the 1% minimum capacity factor) and there is no mechanism to offset emissions when CDR is unavailable. To meet firm capacity requirements in this case, over 600 GW of H2-CTs are deployed along with much smaller quantities (20 GW) of H2-CC, which together provide over 45% of total firm capacity. Given that hydrogen is assumed to cost $2.30 per kg in this case (nearly five times higher, on a $ per energy content basis, than the assumed price of natural gas), the share of generation from hydrogen is less than that in the *NetZero-H2+* case (in which the price of hydrogen is assumed to be approximately $1 per kg). The share of generation from hydrogen in the *GrossZero* case is also lower than the share of unabated natural gas generation in the *NetZero* case for similar reasons. Relative to these two cases, there is more VRE generation in *GrossZero*. However, the H2-CC capacity that deploys is operated at capacity factors significantly higher than observed for the average NG-CC fleet in other cases, more closely resembling how new Gas-CC units are operated in other cases (Figure S16). Results from the sensitivities with the endogenous representation of hydrogen production do not show substantial differences in the role of hydrogen fueled generation resources in meeting energy or firm capacity needs.

Just as hydrogen deploys under certain conditions explored in two of the cases, Gas-CC-CCS makes significant contributions to total generation in two other core cases – the *NetZero-CCS+* case and the *NetNeg* case. In the *NetZero-CCS+* case, more favorable technology and market conditions for natural gas CCS lead to lower relative costs of generation from Gas-CC-CCS, resulting in greater deployment and associated generation. Since Gas-CC-CCS is run at relatively high capacity factors (Figure 2), it primarily competes with other sources of energy, notably VRE. As a result, VRE deployment and the associated generation share (70%) is lower than under the *NetZero* case (82%). Unabated natural gas continues to provide firm capacity, with total unabated natural gas capacity remaining comparable to the other cap cases in which emitting generation is allowed.

In the *NetNeg* case, total generation requirements increase to meet the load associated with DACCS (approximately 1900 TWh in 2050), resulting in the increased deployment of wind, solar, Gas-CC-CCS, and BECCS relative to the *NetZero* case (Figure S17). While the total amount of VRE deployment and generation increase in this case, the combination of the reduction in the quality of remaining wind resources, the declining marginal value of solar, and an earlier phase-out of the ITC and PTC incentives (due to more rapid emissions reductions) lead to a smaller VRE share (76%), with a larger share captured by Gas-CC-CCS, BECCS, and, to a lesser extent, nuclear. In this case, unabated Gas-CC generation declines to 2% of total generation due to higher carbon prices. However, the continued operation of Gas-CC even at the time of net-negative emissions (Figure 2) indicates its value as a peaking energy resource. Finally, because DACCS is assumed to be flexible, it does not increase demand for firm capacity. As such, total firm capacity does not increase along with the overall increase in generation, and total unabated natural gas capacity is comparable to the other cap cases in which emitting generation is allowed.

### Value of electricity system services

**Figure 3:**
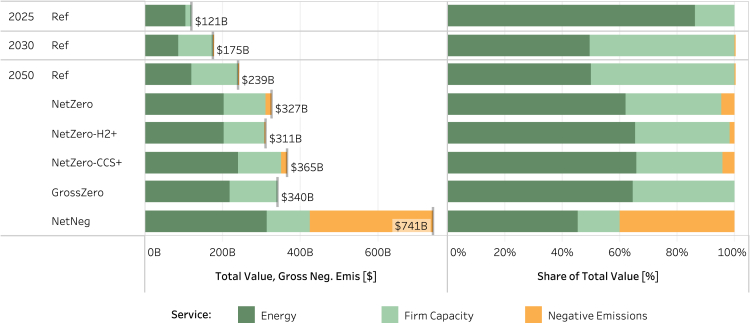
Total value of services and share of total value of services by service type and scenario Results for all scenarios (core and sensitivity scenarios) are shown in Figure S18.

As shown in Figure 3, energy and firm capacity account for almost all service value in most cases. In the *Ref* case, only a small amount of CDR is deployed to help meet stated zero-emissions targets (such as that in California), and in the *Net Zero* cases, there is limited need for CDR given the low utilization of unabated natural gas generation (Figure S16). In the *NetNeg* case, negative emissions account for nearly 40% of total value. Similarly, in the *NetZero-HighDemand* sensitivity (Figure S18), negative emissions comprise a substantial share of value as the increases in demand and changes in the timing of peaking conditions create an increased need for flexible resources, which is met largely with unabated gas capacity, enabled by CDR (Figures S7 and S17).

**Figure 4:**
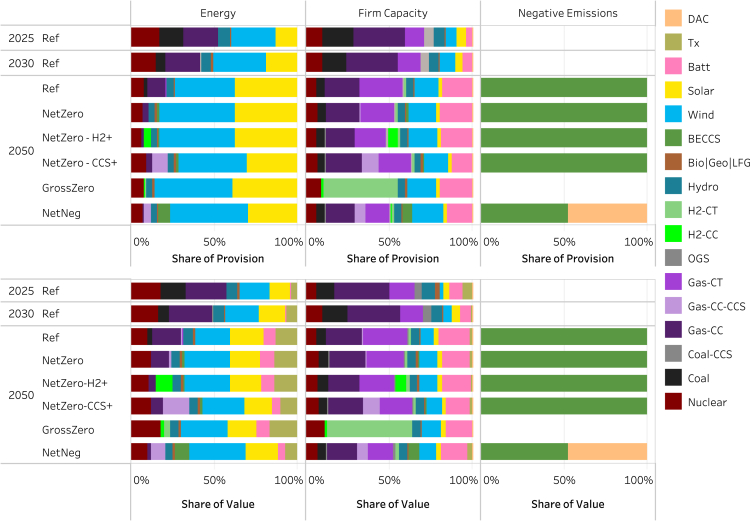
Provision of energy, firm capacity, and negative emissions by technology and scenario Top row shows technology contribution to total service (native units), and the bottom row shows technology contribution to total service value (dollars). For technologies that provide and consume the same service (storage, transmission), net service and net value are shown in this chart. Results for all core and sensitivity cases are shown in Figure S6.

Figure 4 shows the contributions to energy, firm capacity, and negative emissions from each technology by scenario. The top row shows the physical contribution from each technology to the total service, whereas the bottom row shows the shares of total service value. The top-left and top-middle panels correspond to the middle and right panels in Figure 1, except that generation and firm capacity are now shown as shares of the relevant total. Differences between service value and physical service provision arise from price variability over time (time-periods) and/or space (regions). The VRE share of total energy provision is substantially larger than its share of the total energy value (left column). Conversely, dispatchable technologies that account for a smaller share of total generation (particularly unabated natural gas and hydrogen) account for a larger share of total value, highlighting that while these technologies generate less frequently, on average, they do so when electricity prices are higher, during times of greater grid stress (see Figures S1 and S2 and accompanying discussion).

The left column of Figure 4 reveals that two technologies, namely storage and transmission, do not provide positive net energy but do provide positive net energy value. Storage and transmission derive value from energy arbitrage across time and space, respectively. Battery technologies—limited to 4-h and 8-h technologies in these scenarios—are particularly well suited for balancing energy supply and demand on daily timescales (Figure 2), allowing for the increased utilization of VRE, particularly solar PV. Transmission is not limited by duration, but since it can only move power from one region to another, its value is limited by the magnitude of differences in prices, and more fundamentally, resource availability and electricity demand, across regions. Greater (lesser) differences in prices lead to greater (lesser) opportunities for spatial arbitrage.

The middle panels show firm capacity service provision (top) and value (bottom). By 2050, 32–40% of firm capacity is provided by unabated natural gas capacity across net-zero and net-negative cases that allow CDR, and hydrogen technologies comprise over 46% of firm capacity when CDR is disallowed. These large shares of firm capacity provision are achieved even though these technologies account for less than 20% of total capacity in the net-zero cases and even smaller shares of generation. Battery storage accounts for 8–12% of total capacity and 13–20% of firm capacity in net-zero and net-negative scenarios. Unlike natural gas plants, batteries typically receive a lower capacity credit than their total capacity since their availability depends on their state of charge. The VRE contribution to firm capacity is even lower relative to its total capacity, given that the capacity credit of PV declines with increasing share^36^ (see Figure S20). The transmission contribution to firm capacity (both provision and value) is small relative to its contribution to energy. This indicates that it is largely being deployed for its energy value. However, this result could be sensitive to how firm capacity requirements are represented.^38^

The firm capacity results from the sensitivity cases are consistent with the results from the core cases. However, the sensitivities, including the endogenous representation of hydrogen production, demonstrate that flexible load, particularly when it is large and the duration potential is long (as is the case in the *GrossZero-EndogH2 & EconH2* case), can reduce the need for and value of flexible supply-side resources. The share of firm capacity met by combined cycle and combustion turbine resources falls from 46% in the *GrossZero* case to 39% in the *GrossZero-EndogH2 & EconH2* case, and the share of firm capacity value from those technologies falls from 52% to 44%, respectively (Figure S6).

The right panels show the negative emissions provision (top) and value (bottom) provided by BECCS or DACCS in these scenarios. BECCS consistently emerges as the lowest cost CDR option across all cases in which CDR is deployed. As such, in cases in which negative emissions demand is relatively low, as it is in all net-zero cases (Figure S18), BECCS is the only CDR technology deployed. However, in the *NetNeg* case and the *NetZero-HighDemand* sensitivity, the assumed available supply of biomass to the power sector is exhausted, so DACCS effectively deploys as a backstop. Within a given case, the price received per ton removed is the same between BECCS and DACCS, which explains why the shares in the top and bottom panels are identical.

### Technology value

**Figure 5:**
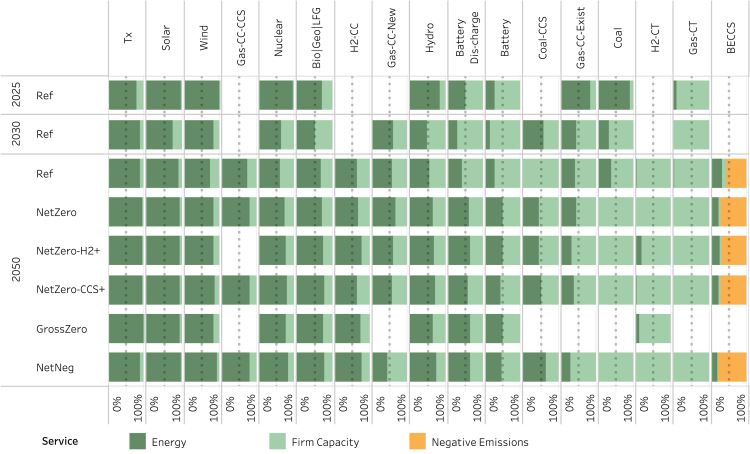
Share of each technology’s total system value by service across scenarios Except for BECCS, technologies are ordered from highest to lowest energy value share under the NetZero case in 2050. For technologies that provide and consume the same service (storage, transmission), net value is shown in this chart, with the gross value for storage shown as a separate column (Battery-Discharge). Gross value of storage is the value associated with discharging (electricity sales), excluding the cost of charging (electricity purchases). The dotted vertical reference lines indicate a 50% share. Results for all core and sensitivity cases are shown in Figure S19, and magnitudes (levels) of values by technology and service are shown in Figure S23.

Figure 5 shows, for each major technology and scenario, the share of value received from the provision of different services. Figure S18 shows the magnitude (levels) of the value by service. Of the technologies that account for most capacity across scenarios in Figure 1 and most value in Figure 4, nine technologies derive a majority of their value from energy: wind, solar, natural gas CCS, nuclear, hydro, H2-CC, New-Gas-CC, other renewables, and transmission (corresponding to the leftmost nine columns in Figure 5). VRE and hydro have relatively low energy production costs and (particularly for VRE) limited contribution to planning reserve margins. In contrast, although natural gas CCS can receive greater capacity credit, it has relatively high capex, which favors investment only when it can run at relatively high capacity factors, which explains its high energy value share. Similarly, existing nuclear capacity has relatively high fixed costs and limited operational flexibility, so it is retained only if it can be operated for much of the year. Finally, transmission derives value primarily from energy arbitrage, with value from capacity only when excess capacity in one region can help meet a deficit in a neighboring region.

In contrast to the technologies above, five other technologies—Coal, Coal-CCS, unabated Gas-CT, existing Gas-CC, and H2-CT (see rightmost five columns in Figure 5 after BECCS)—derive most of their value from firm capacity. These technologies, despite having moderate to higher generation costs, are relatively low-cost options for providing available capacity. In the case of existing resources, which include existing coal, existing Gas-CC, and existing Coal-CCS (which is deployed in earlier years due to the production incentive associated with the §45Q tax credit), investment costs are sunk, so the only costs of maintaining available capacity are the fixed costs. On the other hand, new Gas-CT and H2-CT resources have relatively low capital costs, so they can still be profitable when operated less frequently, despite being generally more expensive to run due to the associated carbon emissions cost (as with natural gas in the cap cases) or high fuel prices (as with hydrogen in most cases).

Among these technologies, however, there are significant differences in cost structure and therefore operation. For example, Gas-CTs (lower capex, higher opex) are only dispatched at very low capacity factors, generally below 1% (Figures 2 and S16). Existing gas-CC units (zero capex, moderate opex) are dispatched at higher levels, with capacity factors ranging from 5 to 10% in the net-zero cases, indicating their value in meeting energy demand during peaking hours. As such, Gas-CTs derive almost all their revenue from capacity, whereas existing gas-CC units derive substantial value from energy provision during peaking hours in the cap cases. For comparison, new gas-CC units (higher capex, lower opex compared to existing units) have annual average capacity factors as high as 22% in the cap cases. This explains why new gas-CC units typically derive a greater share of value from energy while existing gas-CC units derive a greater share of value from firm capacity in the cap cases.

This dynamic, however, does not fully explain the value composition of Coal-CCS. As noted above, a limited amount of Coal-CCS is deployed in earlier years due to the availability of the §45Q tax credit, at which time a substantial share of value is derived from energy (see 2030 in Figure 5). However, by 2050, the tax credit is no longer available, and as a result, generation from Coal-CCS is greatly reduced, tilting its main value stream toward firm capacity.

The value decomposition for battery storage depends to some extent on whether gross energy value or net energy value is considered. Because battery storage derives value from energy arbitrage, energy is both a source of revenue and a cost. When net energy value (energy revenue minus energy cost) is used to decompose value, reflecting the system value of energy, most storage value comes from firm capacity. However, when gross energy value is used to decompose value, reflecting the revenue from selling energy (but not the cost of buying energy), the majority of battery storage value comes from energy in most cases. The latter decomposition demonstrates that while the value of battery storage to the system is dominated by its supply of firm capacity, gross revenue received by storage technologies is dominated by energy. Regardless of the decomposition approach, storage derives substantive value from both energy arbitrage and capacity provision, with the share of value from energy increasing over time in the cap cases, as its utilization increases to balance increasing VRE shares.

A similar accounting issue arises for transmission. However, it is difficult to estimate gross energy value for transmission because gross value scales with the amount of transmission, which depends on the spatial resolution of the model and the associated approach to representing transmission. For this reason, we show only the net energy value for transmission, but in contrast to storage, the energy value still dominates when net energy is used. Transmission expansion across all scenarios is shown in Figure S21.

Negative emissions technologies derive most of their value from negative emissions. While the reason for this is clear in the case of DACCS, which consumes rather than provides energy, it is less so for BECCS, which provides both energy and firm capacity in addition to producing negative emissions. Relative to other generation technologies, BECCS is a higher cost to build and operate. To recover these higher costs, BECCS must receive more revenue than other energy technologies. This additional revenue (value) comes from negative emissions, which lowers the share of value from energy and firm capacity.

Finally, results from the hydrogen sensitivity cases highlight one additional finding, namely that long-duration flexible load, while reducing the value of peaking or firm capacity resources, can also substantially reduce the energy share of total value for flexible technologies that provide a mix of energy and firm capacity. These resources include H2-CC, new and existing Gas-CC, and Coal-CCS. In the *NetZero-EndogH2 & EconH2* case, the increased load associated with electrolytic hydrogen production drives the increased deployment of VRE, but given the flexibility in hydrogen production (given the assumed availability of hydrogen storage), that load can be reduced at times when electricity prices are higher, making more VRE available to incumbent load and reducing the reliance on flexible supply-side resources.^39^

### Connecting technology cost and value

Prior studies have demonstrated that because service prices and, therefore, technology value can vary both spatially and temporally, comparing the levelized cost of electricity (LCOE) is not sufficient to understand how technologies compete and, therefore, what deployment outcomes are expected.^36^^,^^40^^,^^41^^,^^42^^,^^43^ We have shown that, although there is some mixing of roles, technology value can be decomposed into energy provision, firm capacity (and peaking energy), and negative emissions.

Among dispatchable technologies, differences in energy and firm capacity/peaking energy roles are most easily identified by differences in annual capacity factors. Technologies operated at relatively low annual capacity factors typically derive more value from providing firm capacity than from providing energy. Yet, because infrequently dispatched technologies are likely to dispatch when energy prices are high (in times of stress), both primary value streams (firm capacity and energy) are related to the same underlying role—the availability to provide and, in some hours, dispatch peaking energy. For these technologies, the distinction between firm capacity and energy is largely a consequence of the fact that ReEDS represents distinct markets for energy and firm capacity. If the capacity market were eliminated, the cost of capacity would be reflected in energy prices, and these technologies would recover costs by deriving more value from energy in the relatively limited number of operating hours.

For the subset of thermal technologies that are relatively flexible, controlling for differences in capacity factor (as a proxy for role) alleviates the shortcomings of LCOE as a competitiveness metric because total value per unit electricity is similar across technologies. For example, a Gas-CC and a Gas-CT both operating at exactly 5% capacity factor within the same region will dispatch during the top 438 (5% of total hours) energy price-hours in that region and thus receive identical revenue per unit of energy provided. More generally, the flexible thermal technologies dispatched for the fewest number of hours will sell into the market at the highest prices, and the normalized value of energy for flexible technologies will vary inversely with capacity factor (Section SI1).

**Figure 6:**
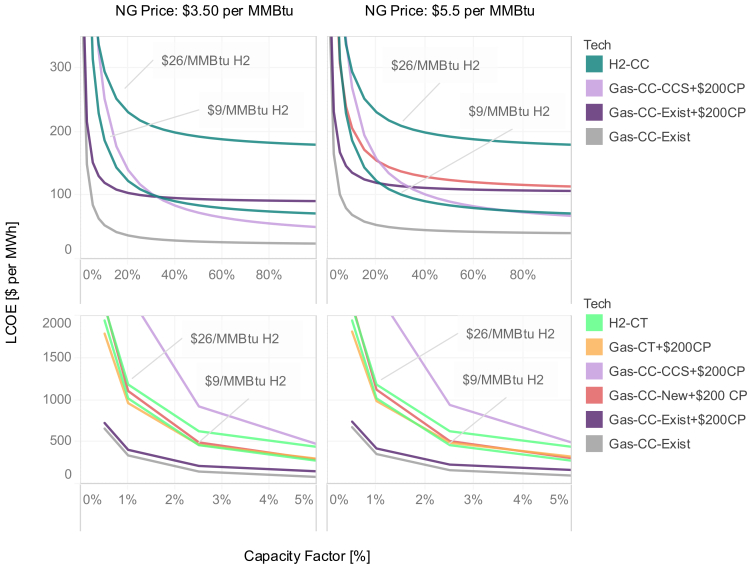
Levelized cost of electricity (LCOE) for natural gas and hydrogen technologies as a function of capacity factor The left column shows LCOEs calculated using assumed natural gas prices of $3.50 per MMBtu, and the right column shows LCOEs calculated using assumed natural gas prices of $5.50 per MMBtu. The top row shows the full range of potential capacity factors and includes firm capacity technologies whose value is composed of a blend of energy and firm capacity value. The bottom row shows a low capacity factor range and (with the exception of Gas-CC-CCS and new Gas-CC) includes technologies whose value is primarily derived from firm capacity provision. LCOEs for hydrogen technologies are calculated using two fuel cost assumptions as indicated by the labels. $26 per MMBtu corresponds to $2.30 per kg, and $9 per MMBtu corresponds to $1 per kg. LCOEs for most gas technologies are shown with a carbon price of $200 per ton CO_2_ ($200CP). Cost and heat rate assumptions used in the LCOE calculations are reported in Table S1. A financial lifetime of 20-year and an interest rate of 5% are also assumed.

Figure 6 shows the LCOE as a function of capacity factor for six of the most flexible technologies: existing Gas-CC, existing Gas-CC retrofitted with CCS, new Gas-CC, new Gas-CT, new H2-CC, and new H2-CT. Existing unabated Gas-CC is shown with and without a carbon price, spanning conditions from the reference and cap cases. H2-CC is shown with the reference hydrogen price as well as with the lower price used for the *NetZero-H2+* case. The left and right panels show the results for different natural gas prices corresponding to reference (left) and low natural gas resource (right) cases.

Under reference natural gas prices and relatively low capacity factors (bottom left of Figure 6), unabated existing natural gas is the lowest cost option (among these thermal technologies) for providing peaking energy, even when facing a $200 per ton carbon price and when zero-carbon hydrogen is available at low cost. This suggests that pairing unabated existing Gas-CC capacity with CDR (at the costs assumed in this study) is often the least cost way of providing firm capacity and peaking energy, even under stringent carbon caps. In the absence of available existing Gas-CC, new Gas-CT is the next most cost-effective option, followed by new gas-CC, although new H2-CT is comparable to new Gas-CT when low-cost hydrogen is available. This explains why new H2-CT is deployed for firm capacity when fossil fuels are disallowed. Notably, Gas-CC-CCS is always the least cost-effective option at low-capacity factors because of its higher fixed costs. The general competition among firm capacity options in the lower panels is not strongly sensitive to the assumed natural gas prices (compare bottom left and bottom right panels).

At higher capacity factors and reference natural gas prices (top left panel of Figure 6), unabated existing gas-CC is most competitive in the reference case. However, when a carbon price comparable to the cap cases is introduced, Gas-CC-CCS is the most competitive option among those considered here. The LCOE for H2-CC is only comparable to the LCOE of Gas-CC-CCS when zero-emissions hydrogen is available at a lower price (demonstrated here at $1 per kg) and when natural gas prices are higher (top right panel of Figure 6). However, at high capacity factors, these technologies compete to provide energy. Thus, as discussed earlier, they are effectively competing with other technologies not shown on this chart, notably VRE, nuclear, and hydroelectric, which explains their relatively modest deployment across the scenarios.

Storage is not shown in Figure 6 because it is not fully flexible, given operational constraints related to its state of charge, and therefore not directly comparable to the other options shown at a given capacity factor. In other words, at a given capacity factor, the hours in which storage is dispatched may be different than the hours in which a flexible thermal technology is dispatched, and therefore the value associated with that dispatch will be different. Notwithstanding these differences, at the capacity factors at which storage is observed to operate in our scenarios (approximately 20% in 2050, as shown in Figure 2, reflecting its use for diurnal balancing), storage is an economic alternative to gas-CC for both daily balancing and firm capacity, particularly when there is a carbon price. On the other hand, storage capital costs are higher than Gas-CT capital costs, which partially explains why storage is not built solely for firm capacity, even though it generally provides and receives a substantial share of total value from firm capacity.

## Discussion

This work evaluates the roles of all major generation, storage, transmission, and negative emissions technologies in a deeply decarbonized U.S. electricity system. Using the ReEDS model, we quantify the provision of key electricity services—energy and firm capacity—by technology and decompose their values (revenues) into energy, firm capacity, and negative emissions across a range of decarbonization scenarios that vary both the stringency of the emissions target as well as key assumptions affecting the competitiveness of technologies. To our knowledge, no study has systematically characterized the value streams of a comprehensive suite of technologies across a range of net-zero to net-negative electricity systems. Addressing these gaps highlights a number of key functional differences between technologies in future electricity systems and the extent to which alternative policies and broader market conditions can alter those roles.

First, consistent with findings from prior research, VRE technologies consistently play an outsized role in supplying energy, while thermal (predominantly unabated natural gas, nuclear, and H2-CC/CT) and storage technologies play an outsized role in supplying firm capacity needed to ensure resource adequacy. VRE technologies comprise 70–85% of total energy generation across our core scenarios, but their share of total energy value is substantially lower (41–54%). This demonstrates that while non-VRE technologies provide a relatively small share of total energy, the value (revenue) associated with that energy is generally comparable to and, under certain conditions, greater than that from VRE. This indicates that non-VRE technologies play a critical role in meeting energy needs cost-effectively.

Second, while the overall deployment of specific technologies is substantially impacted by future market conditions, such as fuel prices and technology costs, their roles—or their value compositions—are largely consistent, regardless of market conditions. VRE, Gas-CC-CCS, and nuclear are, by and large, energy technologies—their value is dominated by energy. As a result, the deployment and total energy shares of these technologies are most closely tied to their relative competitiveness within this technology set. At the other extreme, natural gas and hydrogen combustion turbines are firm capacity technologies—firm capacity provision makes up nearly all of their total value. Between these two extremes are technologies that derive a more balanced share of value from energy and firm capacity, including unbated Gas-CC, H2-CC, and battery technologies. These technologies make substantial contributions to meeting firm capacity requirements, but are also dispatched to meet energy demand during periods of moderate to high prices, leading to capacity factors generally in the range of 10%–25%.

Third, in a net-zero electricity system, it may be cost-effective to retain and potentially expand unabated natural gas capacity. At the time of net-zero, given the relatively low utilization of natural gas capacity, its emissions are sufficiently small that the amount of CDR required to fully offset those emissions would be small relative to total CDR deployment envisioned by many economy-wide net-zero scenarios. Furthermore, although the operational flexibility of natural gas with CCS could be advantageous in a system with a high share of VRE, we find that natural gas with CCS primarily competes with VRE to provide energy rather than with lower capital cost balancing options such as unabated natural gas (paired with CDR) and battery technologies. The higher investment cost associated with natural gas CCS encourages higher utilization rates and discourages deployment as a firm capacity resource. Instead, low capital cost, unabated natural gas is deployed with a small amount of CDR. Although the CDR is relatively high cost, it can be run at high utilization independently from the infrequently dispatched unabated natural gas, effectively decoupling the electricity generation from the carbon capture. As such, we do not see a strong indication that changing the operational flexibility of fossil CCS technologies – or other technologies playing similar roles, such as nuclear – would significantly affect the outcomes observed here.

Fourth, the greatest level of hydrogen fueled generation expansion in the electricity sector occurs in our scenarios when CDR is strictly unavailable, which renders both unabated natural gas and gas with CCS unable to deploy, even as firm capacity. However, in this case, which prohibits fossil, or when hydrogen prices are assumed to be lower, the amount of hydrogen generation is still limited in our scenarios. This occurs because other technologies, such as VRE, as well as existing nuclear and hydro, are typically more competitive in the US. While these conditions and therefore conclusions do not necessarily apply in other world regions, our results suggest that the competitiveness of hydrogen as a generation resource depends on the cost of hydrogen production, transport, and storage, the extent to which fossil generation is permitted under specific net-zero targets, the cost of natural gas, and whether CDR options are available at costs consistent with current projections.

Finally, while technology roles are largely consistent across market conditions explored at the time of net-zero emissions, decarbonization can change how technologies derive value. For example, while existing Gas-CC derives a similar share of value from energy under net-zero scenarios, it does so while dispatching less often but at times of higher prices. Lastly, the extent to which technologies are characterized by a single role may increase, paradoxically increasing the amount of technology complementarity and the importance of technology diversity even as VRE accounts for a greater share of generation. For example, technologies that were historically competitors, such as VRE and unabated natural gas, may become increasingly complementary. Each of these technologies, in turn, may face greater competition with emerging technologies that are not widely deployed today, such as natural gas with CCS (in the case of VRE) or hydrogen (in the case of natural gas).

### Limitations of the study

This study quantifies the value composition of power sector technologies, including generation, storage, transmission, and negative emissions under a range of decarbonized future scenarios. Nonetheless, it does not exhaustively explore potential future conditions. We highlight several limitations.

First, our study focuses on supply-side options for meeting energy and firm capacity requirements. While we evaluate a suite of sensitivities that include new, flexible load from hydrogen production (incremental to the exogenous load captured by the projection used for all cases), we do not capture the opportunity for demand response or increased efficiency associated with incumbent (exogenous) demand to contribute to meeting (or lowering the demand for) electricity services. Increases in load flexibility have the potential to lower the requirement for firm capacity as well as its associated value, and as such could decrease the value of supply side resources providing those services. However, the realized impacts of increased demand side flexibility on the magnitude of firm capacity value would depend on the characteristics of the demand-side resources, and particularly the duration of the available resources. Given that our analysis captures both 4-h and 8-h duration battery technologies, for demand side resources to have a substantial impact on our results, they would need to have lower costs than battery storage or have longer durations. Furthermore, while lower cost, shorter duration (<8 h) demand response could lower the cost of load shifting and reduce energy prices, it may not substantially decrease firm capacity or its associated value, given the growing duration of net-load peaks with increasing load-shifting and the associated duration requirement of load-shifting to reduce peaks.^44^

Second, while we consider alternative end use electricity demands in our sensitivity cases, we do not account for price induced changes in electricity demand, which, given differences in power system costs across these scenarios, could create substantive differences in total demand and hourly demand profiles across scenarios. Such changes could alter the projected values of services, but we do not expect they would substantially change the value composition of individual technologies.

Third, our scenarios with potentially higher electricity demand raise significant questions about the pace of deployment and how real-world constraints, such as those related to siting, may affect competition among options, and by extension, value compositions. Evaluating the sensitivity of results to constraints on the rates of deployment of power sector technologies would be another useful extension of this work.

Finally, in a net-zero energy system, other sectors will compete for several of the same resources, including natural gas, hydrogen, bioenergy, and CO_2_ sequestration. Examining the interaction between sectors at spatial and temporal resolution comparable to what is currently used for electricity expansion is a research frontier. Extending the ReEDS model to include hydrogen and coupling ReEDS to other sectoral models is currently underway and is expected to be an important area of future work.

## Resource availability

### Lead contact

Request for further information and/or resources pertaining to this article should be directed to the corresponding author, Daniel Steinberg (daniel.steinberg@nrel.gov).

### Materials availability

This study did not generate new unique materials.

### Data and code availability

• Data: the input data used to parameterize an open-source version of the model leveraged in this study can be accessed at https://github.com/NREL/ReEDS-2.0.
• Code: an open-source version of the model used for this study is available at https://github.com/NREL/ReEDS-2.0. Modifications to the open-source version were executed for this study, which are not available on the public repository. Parties interested in the code developed for this study can be directed to the corresponding author.
• All other items: requests for other data or information pertaining to this article should be directed to the corresponding author.

## Acknowledgments

The authors thank Dan Bilello, Jaquelin Cochran, Paul Denholm, Trieu Mai, Caitlin Murphy, and Mark Ruth for their helpful comments and input on the article. We also thank Emily Mercer for assistance with editing. This work was authored in part by the National Laboratory of the Rockies, operated by Alliance for Sustainable Energy, LLC, for the U.S. Department of Energy (DOE) under Contract No. DE-AC36- 08GO28308. The NLR authors acknowledge funding support from the ExxonMobil Technology and Engineering Company. The views presented in this article are those of the authors alone and do not necessarily represent those of their institutions or funding entities. The U.S. Government retains, and the publisher, by accepting the article for publication, acknowledges that the U.S. Government retains a non-exclusive, paid-up, irrevocable, worldwide license to publish or reproduce the published form of this work, or allow others to do so, for U.S. Government purposes.

## Author contributions

All authors contributed to the conceptualization of the study, the development of the methods, the analysis, and the development of the article. D.C.S. led the analysis and visualization and co-wrote the article. B.K.M. contributed to the analysis and co-wrote the article. M.M. conducted the modeling runs, data processing, made major contributions to the analysis, and provided critical review and editing of the article. D.P.C. and B.S. contributed to the analysis and provided critical review and editing of the article.

## Declaration of interests

The authors declare no competing interests.

## STAR★Methods

### Key resources table

**Table:** 

REAGENT or RESOURCE	SOURCE	IDENTIFIER
**Deposited data**		

ReEDS Model Data	NLR	https://github.com/NREL/ReEDS-2.0

**Software and algorithms**		

ReEDS Model	NLR	https://github.com/NREL/ReEDS-2.0
GAMS/CPLEX	GAMS Development Corp. GAMS Software GmbH	https://www.gams.com/
Tableau	Tableau from Salesforce	https://www.tableau.com/

### Method details

#### Regional energy deployment system (ReEDS)

We use the ReEDS electricity system capacity expansion and dispatch model to simulate and evaluate the evolution of the US electricity system under a suite of net-zero to net-negative electricity system scenarios.^45^^,^^46^ We subsequently use methods developed in Mowers and Mai^40^ (summarized in Section value of electricity system services) and further applied in other studies^36^^,^^41^^,^^47^ to decompose the system value of technologies that deploy.

ReEDS is a linear optimization model that simulates the evolution of the electricity system by selecting the least-cost mix of electricity generation, storage, and transmission, as well as carbon dioxide management infrastructure, to meet regional electricity demand and policy requirements in the contiguous United States. In this study, ReEDS is configured as a recursive model, solving for an optimal system every five years from the present until 2050. It represents the US grid as a network of 134 balancing areas or zones, within which all physical and policy constraints are enforced.

In each solve-year, ReEDS co-optimizes the investment, retirement, and operation of all generation, storage, transmission, CDR, and CO_2_ transport and storage technologies to meet the suite of grid services (energy, firm capacity, and operating reserves) and environmental requirements (e.g., CO_2_ caps) subject to a range of constraints including resource limitations, and operational constraints of generators and transmission. Services can be met through local provision of resources or through imports from a neighboring region constrained by available transmission capacity.

The model includes a detailed representation of all commercially available electricity generation and storage technologies, as well as technologies at earlier stages of commercialization and deployment. Technologies represented in ReEDS are shown in Table S1. The model allows deployment of new interregional transmission to increase trade of both energy and firm capacity resources, and, in addition, tracks the requirements and costs for intraregional transmission to interconnect wind and solar resources. Interregional transmission can be deployed both within an interconnection and between interconnections at additional cost.

Dispatch of generation and storage resources and operation of the transmission network is captured across 42 representative days with four-hour resolution, for a total of 252 time periods (Both the time resolution and spatial resolution of ReEDS is flexible and can be set by the user. The 2023 Standard Scenarios^46^ uses a nearly identical configuration with 32 representative days). The representative days are selected to capture the spatial and temporal variability in renewable resources (wind and solar) and load patterns over a given year. 33 representative days are selected to characterize more common conditions and associated variability, and nine representative days are selected to capture less frequent, but more extreme load and/or renewable conditions. As such, each of the 365 days within a year are mapped to one of the 42 representative days that most resembles the resource and load conditions within the actual day. The identified dispatch within each representative day is then scaled based on the number of actual days it represents such that the sum of generation in all representative days accounts for all generation over a full year.

In the version of ReEDS used for this study, resource adequacy requirements are represented by enforcing firm capacity constraints—equal to daily forecasted peak load plus a specified planning reserve margin (PRM)—and ensuring that the system has sufficient available ‘firm capacity’ to meet the constraint in all regions and days. The PRM targets are specified for each North American Electric Reliability Corporation (NERC) Assessment Region, with target values taken from the 2023 Long-term Reliability Assessment.^48^ Firm capacity is defined as the product of each generating unit’s nameplate capacity and its capacity credit—the fraction of nameplate capacity available during times of greatest system stress.

While the capacity credit for thermal technologies is assumed to be constant (generally near 100%, but with seasonal adjustments to account for differences in seasonal maximum capacity), the capacity credit of VRE and storage technologies is system dependent—largely a function of the degree of correlation between hourly demand and renewable resource profiles, the generation and transmission network topology, and, in the case of storage, duration. For this reason, the average capacity credit of existing and marginal capacity credit of new VRE and storage resources, by type and region, are calculated and updated between each solve-year.^45^^,^^49^ These calculations, which, for each model-year, leverage regionally resolved and time-synchronous hourly load and renewable resource profiles for seven weather-years, are executed between solve-years with the results passed to the following-year optimization.

The load projections—derived from Evolved Energy Research’s EnergyPATHWAYS model^50^—used in these calculations are produced via (hourly) simulations in which device stock and demand preferences (in a given model year) are identical across weather-year simulations, but temperatures are varied, which in turn, impacts all temperature sensitive loads, including space heating, space cooling, and vehicle charging. The temperature profiles used for those simulations are observed empirical temperatures from 2007-2013. The result is seven 8760-hourly load profiles in which each day is mapped to a representative day used in the model’s optimization.

The capacity credit for VRE technologies is assigned for every representative day and region as the available capacity (capacity factor) of the relevant technology in the single highest net-load hour across all corresponding hours within the 7 weather-year profiles. Further details and graphical representations of this method can be found in Frew et al.^51^ and in the ReEDS online documentation (Available at https://pages.github.nrel.gov/ReEDS/ReEDS-2.0/index.html). The capacity credit of storage resources is assigned based on the ratio of the duration of a storage resource to the estimated duration required to achieve ‘full’ capacity credit, a capacity credit of 1. This approach accounts for the increasing storage duration required to reduce net-load peaks with increasing deployment of storage.^44^

Relative to the 2023 open-source version of ReEDS used in Gagnon et al.,^46^ the version implemented here incorporates several modifications. First, we enable the endogenous representation of CO_2_ transport and storage (CTS) infrastructure investment and operation. By default, ReEDS applies a uniform cost to any CO_2_ capturing technology to account for the cost of transport and long-term geologic storage. The CTS module,^37^ instead, specifies the spatial extent, costs of development, and physical characteristics of saline storage formations, the costs and operational constraints of transmission pipelines, and requirement that sufficient infrastructure is deployed and operated in each time period to transport, inject, and store all captured CO_2_ .

Second, we remove the minimum annual capacity factor requirement for all thermal generators, set at 6% by default in ReEDS. This requirement, which is more consequential in ReEDS simulations with lower temporal resolution, is intended to capture the historical behavior of the Gas-CT fleet, as well as the expectation that any online unit, even if largely maintained to provide firm capacity, would dispatch for some number of hours. However, under net-zero to net-negative emissions targets, it may be optimal to maintain emitting plants largely for their firm capacity contributions and for energy during times of more extreme system stress. Such plants would be operated at lower capacity factors, avoiding costs associated with emissions that would result from higher utilization. As such, we remove the minimum capacity factor constraint in this study (except in the case without CDR), allowing individual units to remain online without dispatching if it is economic to do so. However, as shown in the results section, all remaining capacity dispatches to some extent. In addition, we report results from additional simulations that include a minimum capacity factor constraint set at 1% and demonstrate that this constraint does not significantly affect results in the *NetZero* case described below (Figure S3).

Third, in order to avoid the complex interactions between hydrogen and electricity production in our core scenarios—particularly given the interactions of tax credit incentives available for clean hydrogen production through both §45V and §45Q of the US tax code and the tax credits for clean electricity—we simplify the representation of hydrogen supply for electricity generation by assuming a fixed price of hydrogen available to hydrogen combustion turbines and combined cycle generators in the electricity sector. However, we enable an endogenous representation of the hydrogen production sector in sensitivity cases based on a limited number of core scenarios. These cases capture the investment in and operation of hydrogen production facilities to meet existing and new demands for hydrogen within both the power sector and the rest of the economy.

Lastly, while cost and performance for most technologies are sourced from the 2023 Annual Technology Baseline,^52^ assumptions for fossil, hydrogen, and CCS technologies are based on Brown et al.,^37^ as discussed further in Section technology value.

#### Technology value in ReEDS

We focus on the three most valuable services in a net-zero electricity system: energy, firm capacity, and negative emissions (ReEDS accounts for other grid services and policy-related constraints, including operating reserves and state policies such as renewable energy and clean energy standards, as well as state cap-and-trade policies. However, given that energy, firm capacity, and negative emissions account for most of the total value, we focus on those three services here). Within ReEDS, all electricity service markets are captured through a balancing constraint, which requires that the supply of each service is equal to (or exceeds) total demand for that service. When that constraint is binding, its shadow price represents the marginal cost of service provision. This price is equivalent to the market clearing price for the service in a competitive market. Multiplying this price by the quantity of service provided (by region and time slice) gives the total service value, and similarly represents the total (net) revenue received for a service. Some technologies, such as storage and transmission contributions to energy, can have both positive (sales) and negative (purchases) revenue in a single service market.

Energy prices reflect the long-run marginal cost of producing electricity when supply and demand are balanced in each time-period. Capacity prices reflect the capacity market clearing price determined from the shadow price on the planning reserve margin constraint. Emissions prices reflect the marginal CO_2_ abatement cost, or equivalently the cost of CO_2_ allowances in a tradable emissions permit market. Under the cap cases, emitting generators must purchase sufficient emissions allowances to cover their annual emissions, thereby incurring an emissions cost, whereas negative emissions technologies such as BECCS or DACCS generate emissions allowances by removing carbon from the atmosphere. Because emissions allowances can be sold, BECCS and DACCS effectively receive payment for negative emissions, and because our analysis compares value, not cost, across services and technologies, we focus on negative emissions (CO_2_ removal) as the relevant emissions-related service.

Following methods detailed by Mowers and Mai,^40^ the value of each technology is decomposed into its components in a given model year as follows:

$$V_{T}=\sum_{S,r,t}Q_{T,S,r,t}*P_{S,r,t}$$

where $V_{T}$ is annual value attributed to technology T, $Q_{T,S,t,l}$ is the quantity of service S (either energy, firm capacity, or emissions reductions) provided by technology T in region r at time-period t, and $P_{S,r,t}$ is the price of service S in region r at time-period t. The service prices reported are the shadow values on the respective model constraints.

Distinguishing between ‘energy’ and ‘firm capacity’ value is most straightforward in an electricity market (or in an electricity capacity expansion model) with a long-run capacity market. Energy-only electricity markets do not have explicit capacity markets and thus do not have capacity payments. Instead, they rely on scarcity pricing of energy to compensate generators operating during times of higher-than-anticipated demand or lower-than-anticipated generator availability. In this type of market, firm capacity value would be embedded in the energy value. Distinguishing between sources of value would therefore require categorizing and separating energy value derived during times of system stress (firm capacity value) from energy value derived at other times (energy value).

Despite this, most long-term capacity expansion or planning tools used by utilities in their integrated resource planning process, distinguish energy and firm capacity requirements. The disaggregation of value streams in this study can therefore be used to inform utilities, system operators, and other relevant decision-makers about how different assets could be used and how they could derive value.

#### Scenarios

We simulate a suite of scenarios that vary the national CO_2_ emissions target, the availability, cost, and performance of key generation and storage technologies, and fuel prices for natural gas and hydrogen. We evaluate four different emissions targets: (1) *Reference*, a no new policy case that does not include an emissions target; (2) *NetZero,* a cap achieving *net*-zero emissions by 2050, allowing the use of CDR technologies to offset remaining emissions from the power sector; (3) *GrossZero*, a cap achieving *gross-*zero emissions by 2050 that assumes CDR technologies are unavailable and requires a minimum capacity factor of thermal technologies of 1%, thereby prohibiting generation, capacity, and operating reserve provision from emitting technologies in 2050; and (4) *NetNeg*, a cap that achieves *net-negative* one gigaton (1 Gt) CO_2_ emissions by 2050 (and net-zero emissions by 2038), reflecting a net-zero energy system in which the power sector provides negative emissions to offset remaining gross emissions in other sectors. 1 Gt of negative emissions is broadly consistent with the gross-negative emissions levels at the time of net-zero emissions identified in other studies of energy-system decarbonization (e.g., Binsted et al. 2024, Williams et al. 2021^25^^,^^53^).

Two variants to the *NetZero* scenario are simulated, one in which future conditions are more favorable to hydrogen generation (*NetZero-H2+*) and another in which future conditions are more favorable to fossil generation with CCS (*NetZero-CCS+*). The former assumes lower hydrogen prices and higher natural gas prices, and the latter assumes conservative cost and performance characteristics (limited improvement) for batteries and renewables, advanced cost and performance characteristics (rapid improvement) for CCS technologies, and low natural gas prices. The suite of scenarios analyzed in this work is summarized in Table 1.

All cases include a representation of the Inflation Reduction Act (IRA) incentives for electricity sector technologies, including a dynamic phaseout of the §45Y production tax credit (PTC) and §48E investment tax credit (ITC) incentives for zero carbon emitting resources based on realized grid emissions. The §45V incentive for clean hydrogen production is not included, but the *NetZero-H2+* case assumes lower hydrogen prices that could reflect, in part, the impacts of policy. In the Reference Case, the electricity sector incentives do not phase out by the end of the simulation (2050), but in the other cases, the phaseout is triggered prior to 2050, with the trigger reached earliest in the net-negative case. Emissions trajectories are shown in Figure S4.

Reference fuel price projections for natural gas, coal, and nuclear are from the EIA 2023 Annual Energy Outlook (AEO23) *Reference* case.^54^ The AEO23 *High Oil and Gas Resource* (AEO23-HOG) and *Low Oil and Gas Resource* (AEO23-LOG) cases are used for the low natural gas price and high natural gas price projections, respectively (See Figure S5). Zero-emissions hydrogen is assumed to be available to the power sector at a fixed price of $20 per MMBtu (approximately $2.30 per kg) in all years under reference conditions and at $9 per MMBtu (approximately $1 per kg) under “low price” conditions. Biomass supply to the power sector is represented as regional supply curves derived from DOE2016.^55^ Biomass supply is limited to available woody biomass with total national supply of 116 million dry tons (or approximately 1.5 quads). However, under the *NetNeg* case, we assume there is five-times more biomass available at each price than under the default assumption to reflect a scenario in which substantially more negative emissions are required from the power sector to achieve an economy-wide decarbonization target. While this level of biomass utilization in the power sector may be unlikely, this scenario was included to explore how BECCS would derive value if it were to deploy in large quantities.

Cost and performance assumptions for generation and storage technologies are from the Annual Technology Baseline 2023,^52^ *Moderate* and *Conservative* cases, with the exception of fossil, fossil-CCS, and hydrogen generation technologies, as well as CDR technologies, which are based on Brown et al.^37^ Low and high CO_2_ capture rate technologies are represented for both Gas-CC-CCS (90% and 97% capture) and Coal-CCS (90% and 99%) technologies. CCS retrofits are allowed for all unabated gas and coal plants. Capital costs for retrofits are assumed to be equivalent to the difference in cost between a unit with and without a capture system scaled upward by 20% to account for additional costs associated with retrofits relative to greenfield builds. Cost and performance assumptions for all technologies for which assumptions are not derived from the Annual Technology Baseline are reported in Tables S2 and S3.

Electricity consumption for non-DACCS demand is from the NLR 2023 Standard Scenarios-Mid Case^46^ and is assumed to be identical across all core scenarios, rising from just over 4,000 TWh in 2025 to approximately 6,500 TWh in 2050, accounting for load growth due to accelerating electrification and population growth, among other drivers. Electricity demand from DACCS is endogenous. As such, electricity load exceeds the reference projection in the *NetNeg* case in which DACCS is deployed and operated. While the evolution of load may differ depending on electricity prices, given substantial uncertainty in the evolution of load under the scenarios explored here, we use a harmonized projection of load to isolate the impacts of non-load drivers on technology provision and value. However, to test the sensitivity of our results to changes in demand, we evaluate a sensitivity to the *NetZero* case—the *NetZero-HighDemand* case—in which exogenous load rises at a substantially higher rate leading to annual load in 2050 of over 11,000 TWh. This projection assumes a high degree of electrification of transportation and buildings space heating, leading to changes in load profiles, including increases in the magnitude and timing of peaks.

Finally, we simulate two additional suites of sensitivities to our core *Reference*, *NetZero*, and *GrossZero* cases in which we include an endogenous representation of investment in and operation of hydrogen production capacity to meet demand for hydrogen in both the power sector and rest of economy. These sensitivities were included to evaluate how electricity demands associated with hydrogen production would impact our core findings. In the first suite, hydrogen supply is required to meet endogenous hydrogen demand from only the power sector, denoted as *‘EndogH2’*; in the second suite, hydrogen supply is required to meet endogenous hydrogen demand from both the power sector and the rest of the economy (ROE), which is specified exogenously, denoted as ‘*EndogH2 & EconH2*’. The projected demand for hydrogen in the rest-of-economy—which reaches 46 Mtonnes per year by 2050—is taken from the ‘Accelerated Demand Electrification’ scenario from Denholm et al.^9^ and represents a scenario in which increased reliance on clean hydrogen as both an intermediate and final energy source plays a significant role in achieving emissions reductions across the economy.

Our figures and discussion focus on results from the core scenarios, with results from all scenarios and sensitivities provided in the supplemental information.

### Quantification and statistical analysis

Quantification, analysis, and visualization of results from the model simulations were carried out using a combination of Python, Microsoft Excel, and Tableau. Details of the methods used to aggregate data represented in all figures are specified in the method details section or in the figure captions.
