# Supplementary material for: Decomposing sources of value for electricity and negative emissions technologies in net-zero power systems
Source: iScience. 2025 Dec 9;29(3):114187. doi: 10.1016/j.isci.2025.114187 (PMC12969129; doi:10.1016/j.isci.2025.114187)
Supplement: Document S1. Figures S1–S25 and Tables S1–S3 [file mmc1.pdf]

iScience, Volume *29*

Supplemental information

Decomposing sources of value for electricity and negative emissions technologies in net-zero power systems

Daniel C. Steinberg, Daniel P. Cherney, Bryan K. Mignone, Matthew Mowers, and Brian Sergi

Supporting Information

SI 1. Normalized value of energy

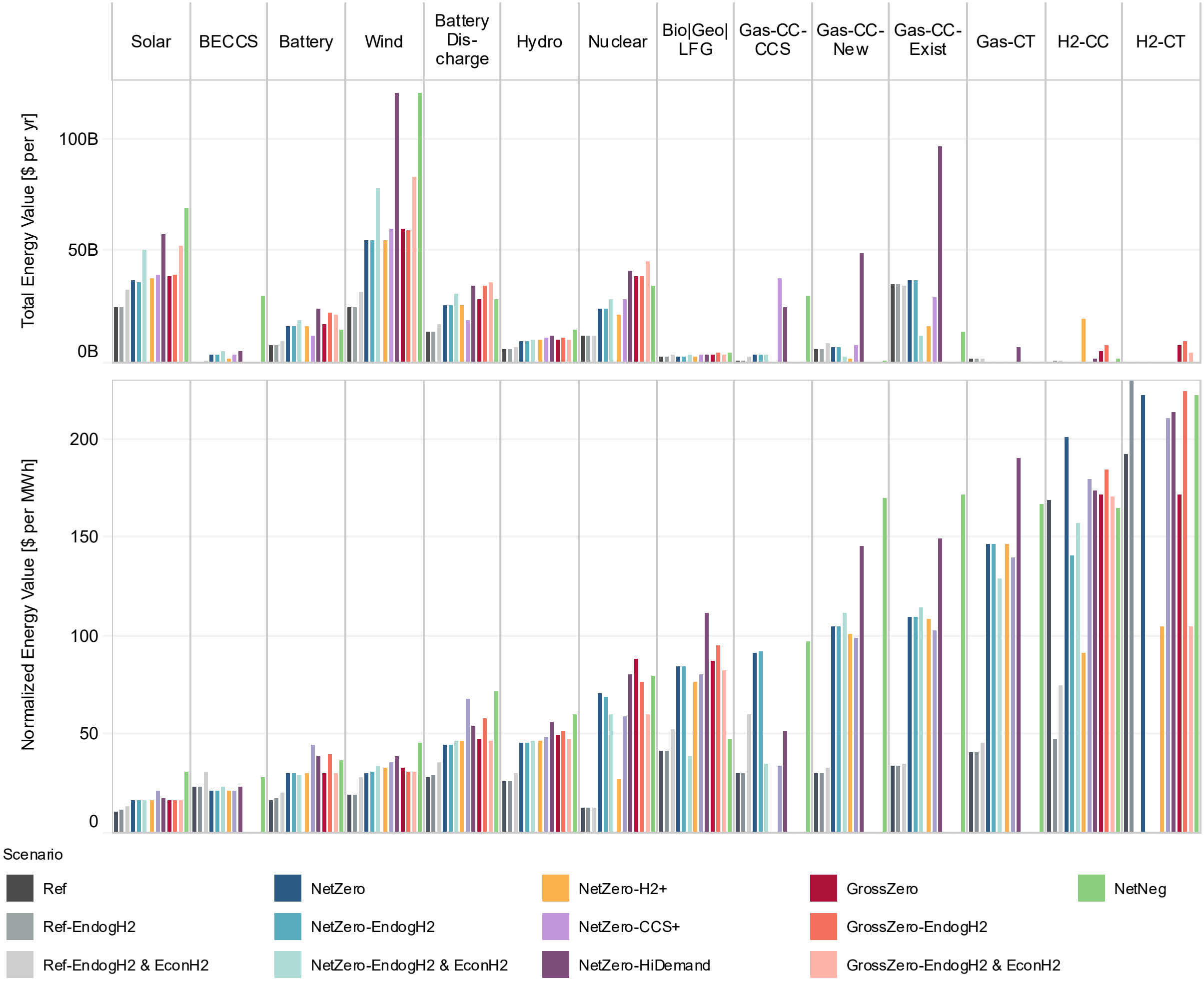

Figure S1. Total energy value (top row) and normalized energy value (bottom row) by technology and scenario in 2050 ordered by the normalized energy value in the NetZero scenario. Values of energy for storage are shown using both net value (column labeled ""battery"), reflecting the system value of energy provision from batteries after accounting for energy purchased for charging, and gross value (column labeled "Battery Discharge"), reflecting the gross energy sales revenue received by batteries (but excluding the cost of energy purchases for charging).

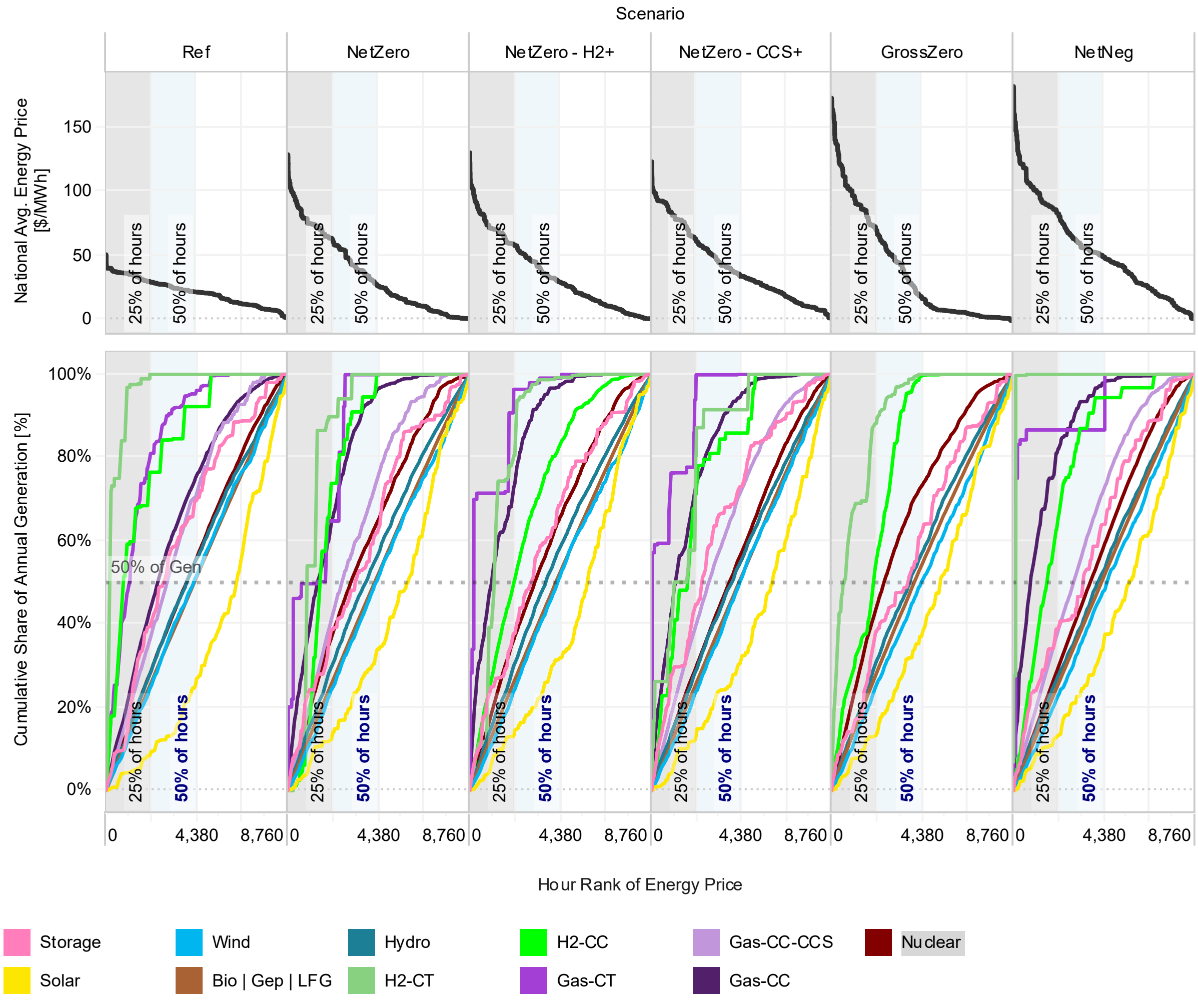

Figure S2. Cumulative share of generation in 2050 for each technology over hours ranked from highest price to lowest price within each of the core scenarios. Not all technologies are shown to improve readability.

While decomposing electricity system services into energy, firm capacity, and emissions provides insights about the roles of different technologies in a net-zero electricity system, these aggregate metrics do not reveal important differences in the temporal patterns of service provision, which is particularly relevant for energy. Specifically, some technologies provide energy when it is more valuable (prices are higher), while other technologies provide energy when it is less valuable (prices are lower). In general, the same total value could be attained by generating less in periods of higher average prices or generating more in periods with lower average prices.

Figure S1 shows the normalized value by technology and scenario. The normalized value for VRE is among the lowest of technologies shown. This is driven by the fact that the VRE marginal cost of generation is zero, and as a result, greater shares of VRE generation in a given period generally reduce the market clearing price for energy. Yet, despite the lower normalized value, the total energy value of VRE exceeds all other technologies, due to the amount of energy provided by VRE.

In contrast, in most scenarios, unabated natural gas and hydrogen dispatch infrequently but do so when energy is scarce and therefore valuable. Other dispatchable technologies, including nuclear, natural gas CCS, and hydroelectric operate at higher capacity factors (50-85%), leading to lower normalized energy values. Finally, while battery capacity factors are relatively low, generally around 20% (Figure S16) across the cap cases, their operation is not fully flexible (it is dependent on the duration and state of charge of the battery), and thus battery storage cannot necessarily provide energy during all times of greatest system value (and associated higher prices).

Differences in energy value can also be observed in Figure S2, which shows the cumulative generation for a subset of technology categories across hours ordered by energy price in each time slice. In the cap cases, H2-CT, Gas-CT, and Gas-CC exhibit the most concave curves, which are steepest initially, indicating that most of their generation occurs during the top 25% of energy price hours. For Gas-CT and Gas-CC, the initial slope and concavity is higher in the cap cases than in the *Ref* case, indicating a greater concentration of generation in higher price hours, with the most pronounced effect in the *NetNeg* case in which over 75% of the total generation of Gas-CC is within the top 25% of energy price hours. H2-CC generally has a somewhat lower slope initially than Gas-CC, given generally higher capacity factors and generation during lower energy price hours than the average Gas-CC fleet, with the effect most pronounced in the two scenarios most favorable to hydrogen generation—the *NetZero-H2+* and *GrossZero* case. The curves for VRE are the most convex, with lower initial slopes, indicating that a substantial share of generation occurs during the lowest 50% of energy price hours. Consistent with Figure S1, the curves for Gas-CC-CCS and batteries fall in between the others, suggesting that they derive more energy value in high price periods than VRE, but more energy in low price periods than unabated natural gas or hydrogen.

SI2. Additional tables and figures

Table S1. Electricity technologies included in the ReEDS model.

Technology Label	Description
**DACCS**	Direct air carbon dioxide capture and storage (sorbent based, electricity powered)
**Tx**	Transmission
**Storage**	Batteries (four and eight hour) and pumped-hydropower
**Solar**	Utility-scale PV, distributed rooftop PV (exogenous [a]), and concentrating solar power
**Wind**	Land-based and offshore wind
**BECCS**	Biopower with carbon capture and storage
**Bio**	Biopower
**Geo**	Geothermal
**LFG**	Landfill gas
**Hydro**	Hydropower
**H2-CT**	Hydrogen combustion turbine
**H2-CC**	Hydrogen combined cycle
**OGS**	Oil-gas-steam
**Gas-CT**	Natural gas combustion turbine
**Gas-CC**	Natural gas combined cycle
**Gas-CC-CCS**	Natural gas combined cycle with carbon capture and storage (options for 90% and 97% CO_2 capture), greenfield and retrofit
**Coal**	Pulverized coal
**Coal-CCS**	Coal with carbon capture and storage (options for 90% and 99% CO_2 capture), greenfield and retrofit
**Nuclear**	Light water reactor, small modular reactor

[a] Distributed PV capacity is assumed to reach 173 GW, nationally, by 2050, accounting for 283 TWh of generation.

Table S2. Cost and performance assumptions for generation technology assumptions not taken from the 2023 Annual Technology Baseline[48].

	Capital Cost [2020$ per kW]			Fixed O&M [2020$ per kW-yr]			Non-fuel Variable O&M [2020$ per MWh]			Heat Rate [MMBtu per MWh]		
	**2025 (Mod)**	**2050 (Mod)**	**2050 (Adv)**	**2025 (Mod)**	**2050 (Mod)**	**2050 (Adv)**	**2025 (Mod)**	**2050 (Mod)**	**2050 (Adv)**	**2025 (Mod)**	**2050 (Mod)**	**2050 (Adv)**
**BECCS-90% capture**	5,768	3,953	-	163	145	-	14.92	12.77	-	11.9	10.9	-
**BECCS-99% capture**	6,000	4,112	-	163	145	-	16.02	13.71	-	12.7	11.6	-
**H2-CT**	820	705	-	21	21	-	4.94	4.94	-	9.7	9.7	-
**H2-CC**	1,105	984	-	28	28	-	1.74	1.74	-	6.4	6.4	-
**Gas-CT**	746	641	-	21	21	-	4.94	4.94	-	9.7	9.7	-
**Gas-CC**	858	764	-	28	28	-	1.74	1.74	-	6.4	6.4	-
**Gas-CC-CCS-90% (greenfield)**	2,519	1,734	1,369	66	51	40	5.78	4.75	3.87	7.2	6.9	6.6
**Gas-CC-CCS-97% (greenfield)**	2,602	1,792	1,413	68	52	42	6.07	4.99	4.06	7.3	7	6.7
**Gas-CC-CCS-90% (retrofit)**	1,993	1,164	726	66	51	40	5.78	4.75	3.87	7.2	6.9	6.6
**Gas-CC-CCS-97% (retrofit)**	2,093	1,234	779	68	52	42	6.07	4.99	4.06	7.3	7	6.7
**Coal**	2,337	1,871	-	73	70	-	7.8	7.35	-	8.5	7.8	-
**Coal-CCS-90% capture (greenfield)**	4,578	3,214	2,927	123	107	96	14.36	12.33	10.8	10.8	10	9.2
**Coal-CCS-99% capture (greenfield)**	4,795	3,366	3,064	128	111	100	15.33	13.16	11.56	11.4	10.5	9.7
**Coal-CCS-90% capture (retrofit)**	2,689	1,612	1,267	123	107	96	14.36	12.33	10.8	10.8	10	9.2
**Coal-CCS-99% capture (retrofit)**	2,950	1,794	1,432	128	111	100	15.33	13.16	11.56	11.4	10.5	9.7

Table S2. Cost and performance assumptions for electricity consuming technologies, including sorbent-based direct air capture and CO_2 storage (DACCS), and steam methane reforming (with and without CCS) and electrolytic hydrogen production technologies.

	**Capital Cost** DACCS: [2020$ per (t CO_2/yr)] H_2 Techs: [2020$ per kW-equiv]		**Fixed O&M** DACCS: [2020$ per ((t CO_2/yr)*yr)] H_2 Techs: [2020$ per (kW-equiv*yr)]		**Non-fuel Variable O&M** DACCS: [2020$ per t CO_2] H_2 Techs: [2020$ per kg]		**Electricity Use** DACCS: [MWh per t CO_2] H2: [kWh per kg]		**Natural Gas Use** H2: [MMBtu per kg]	
	**2025**	**2050**	**2025**	**2050**	**2025**	**2050**	**2025**	**2050**	**2025**	**2050**
**DACCS**	942	706	35	34	46.7	45.5	4.4	4.3	-	-
**SMR**	557	534	17.8	17.2	0.087	0.087	0.9	0.9	0.192	0.192
**SMR-CCS**	1161	1064	37.3	34.2	0.089	0.089	1.9	1.9	0.192	0.192
**Electrolyzer**	1300	550	65	27.5	0.0	0.0	56.1	51.5	-	-

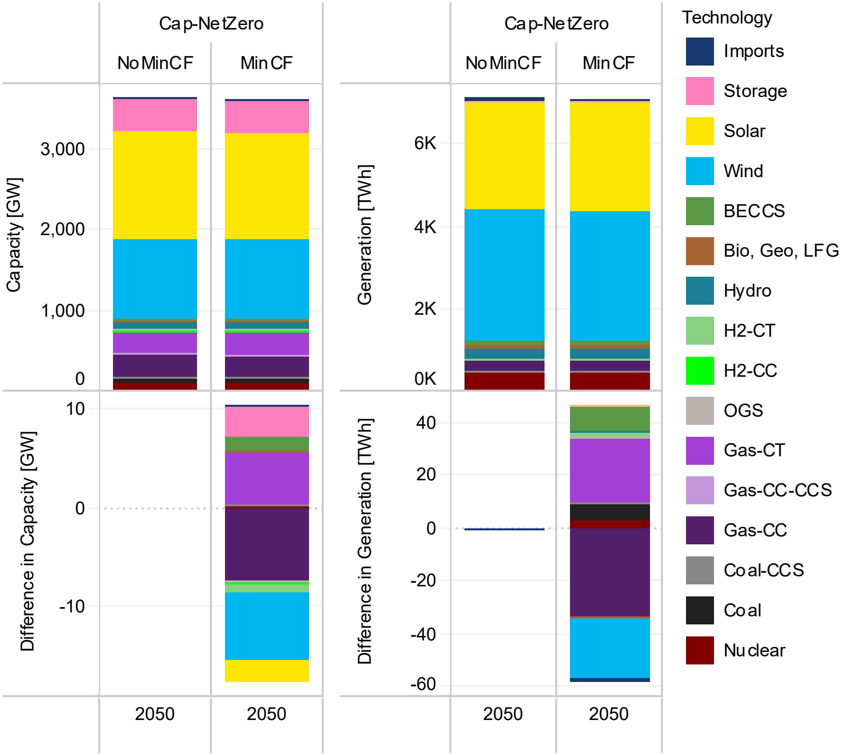

Figure S3. Capacity (left) and generation (right) in the NetZero case excluding and including a 1% minimum capacity factor constraint (MinCF). The top row shows the levels and the bottom row shows the difference from the NoMinCF cases. Inclusion of the minimum capacity factor has negligible impacts on capacity and generation results in this case.

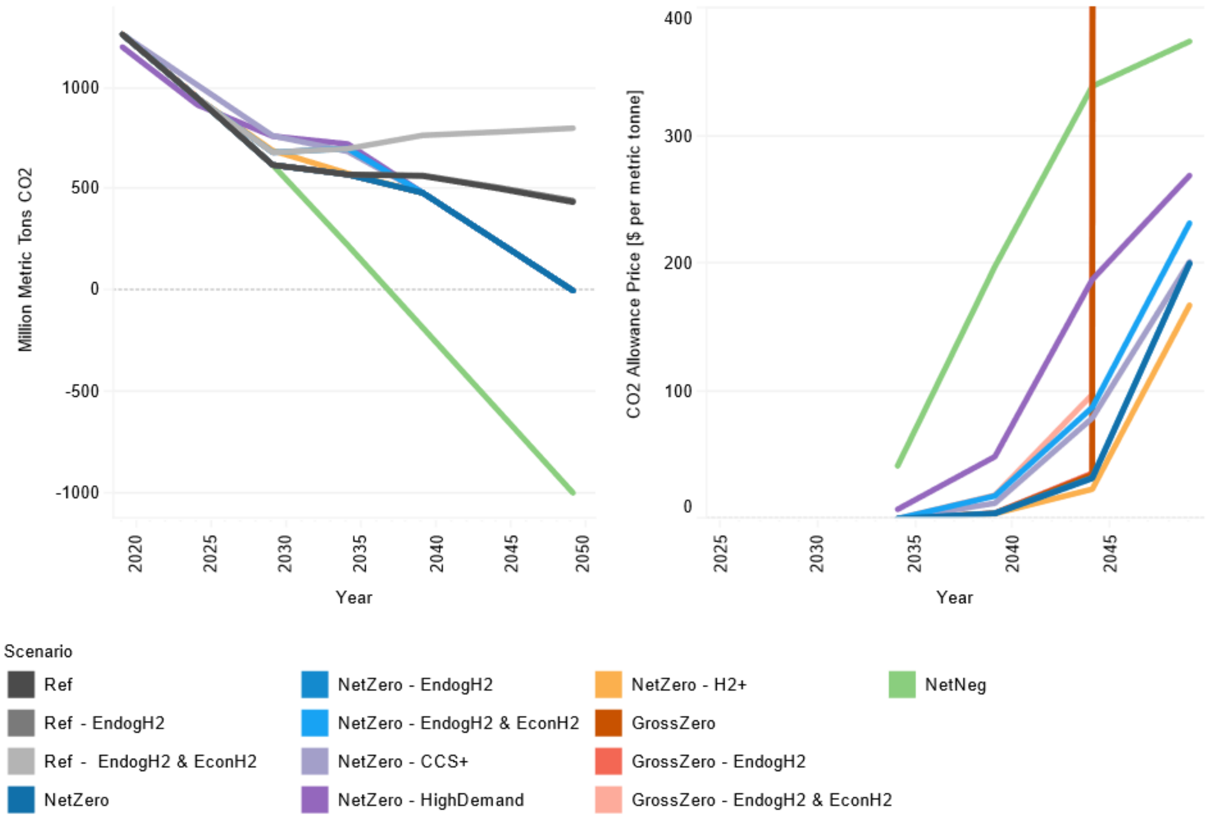

Figure S4. Power sector emissions and the marginal CO2 emissions allowance price by year and scenario. Under the GrossZero scenario the marginal allowance price spikes in 2045 due to the need to invest in a large amount of zero-emitting capacity in order to offset relatively few remaining emissions. The chart is truncated at $400 per tonne CO2.

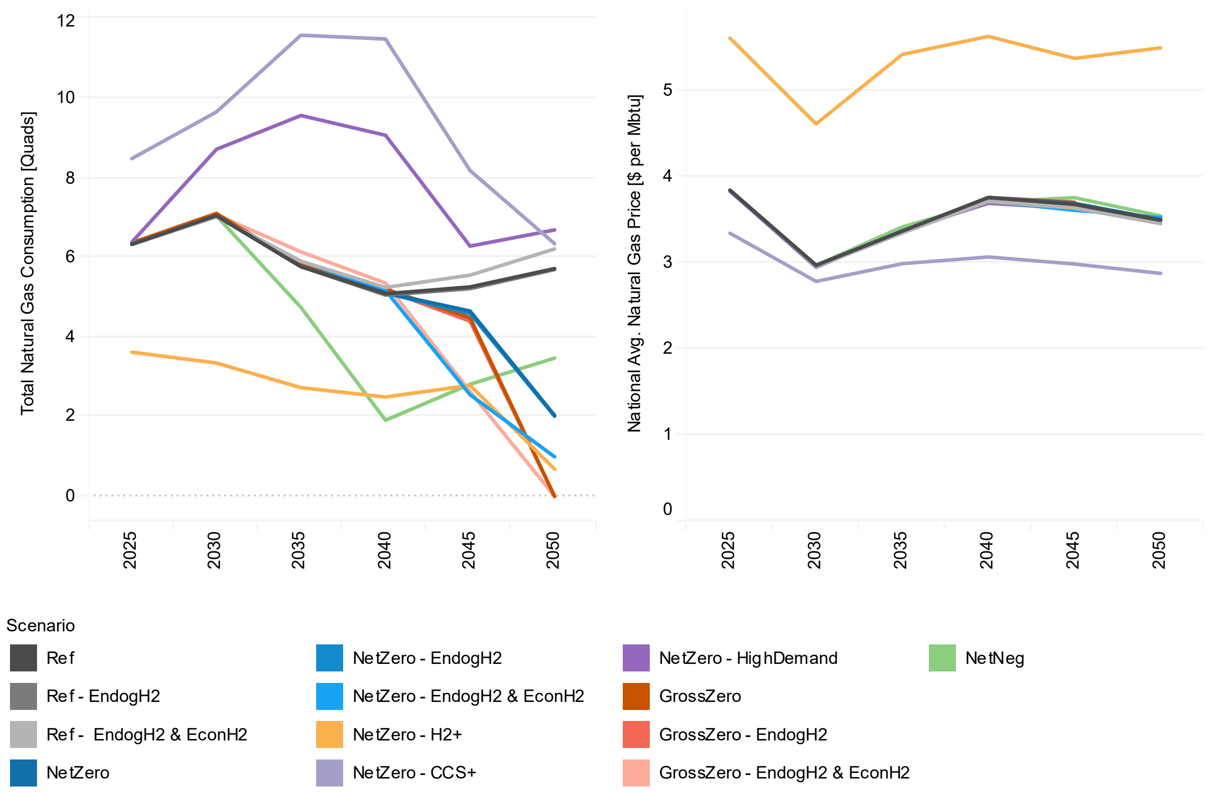

Figure S5. Natural gas consumption (left) and price (right) by year and scenario.

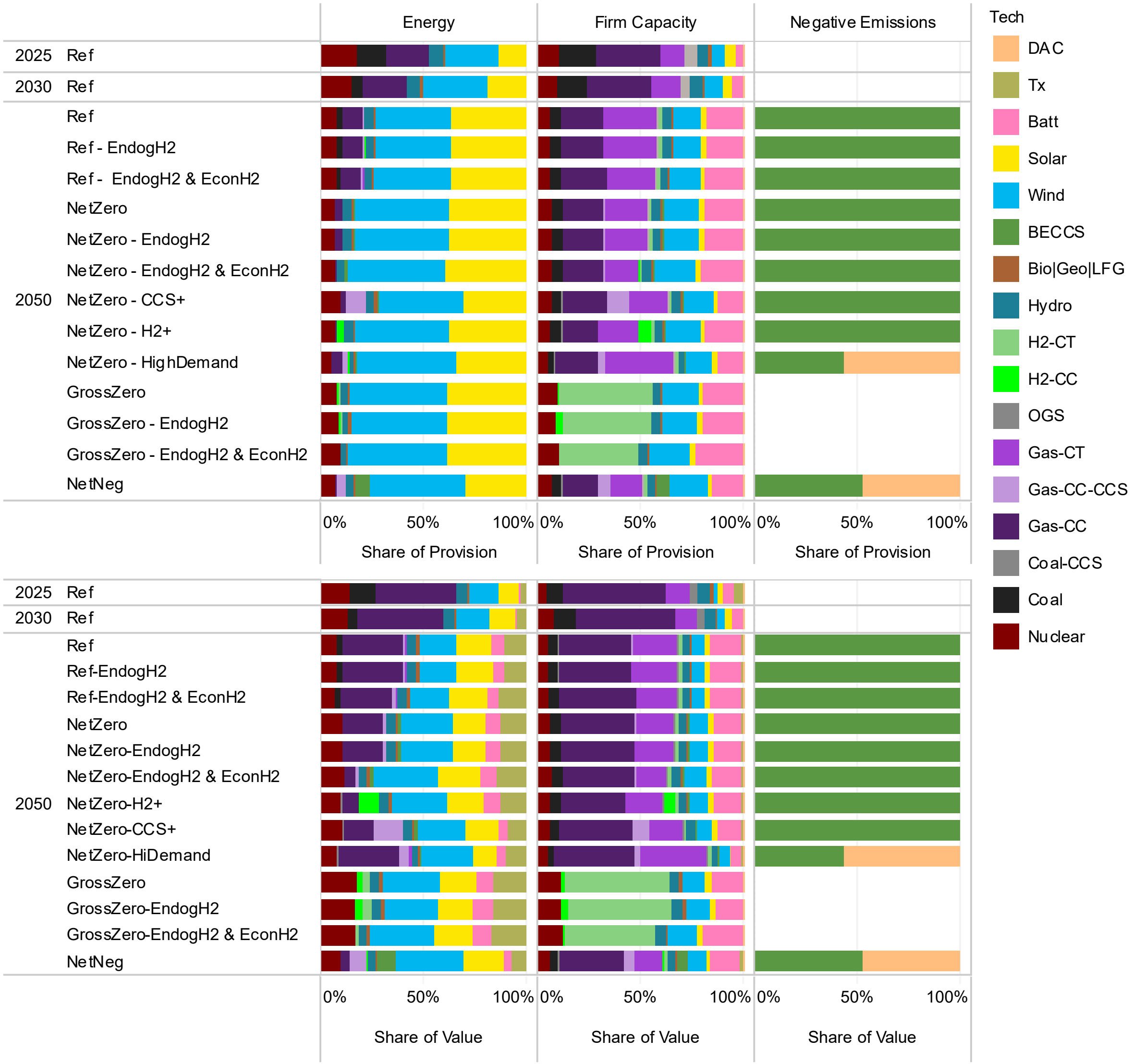

Figure S6. Provision of energy, firm capacity, and negative emissions by technology and scenario in 2050. Top row shows technology contribution to total service (native units), and bottom row shows technology contribution to total service value (dollars). For technologies that provide and consume the same service (storage, transmission), net service and net value are shown in this chart.

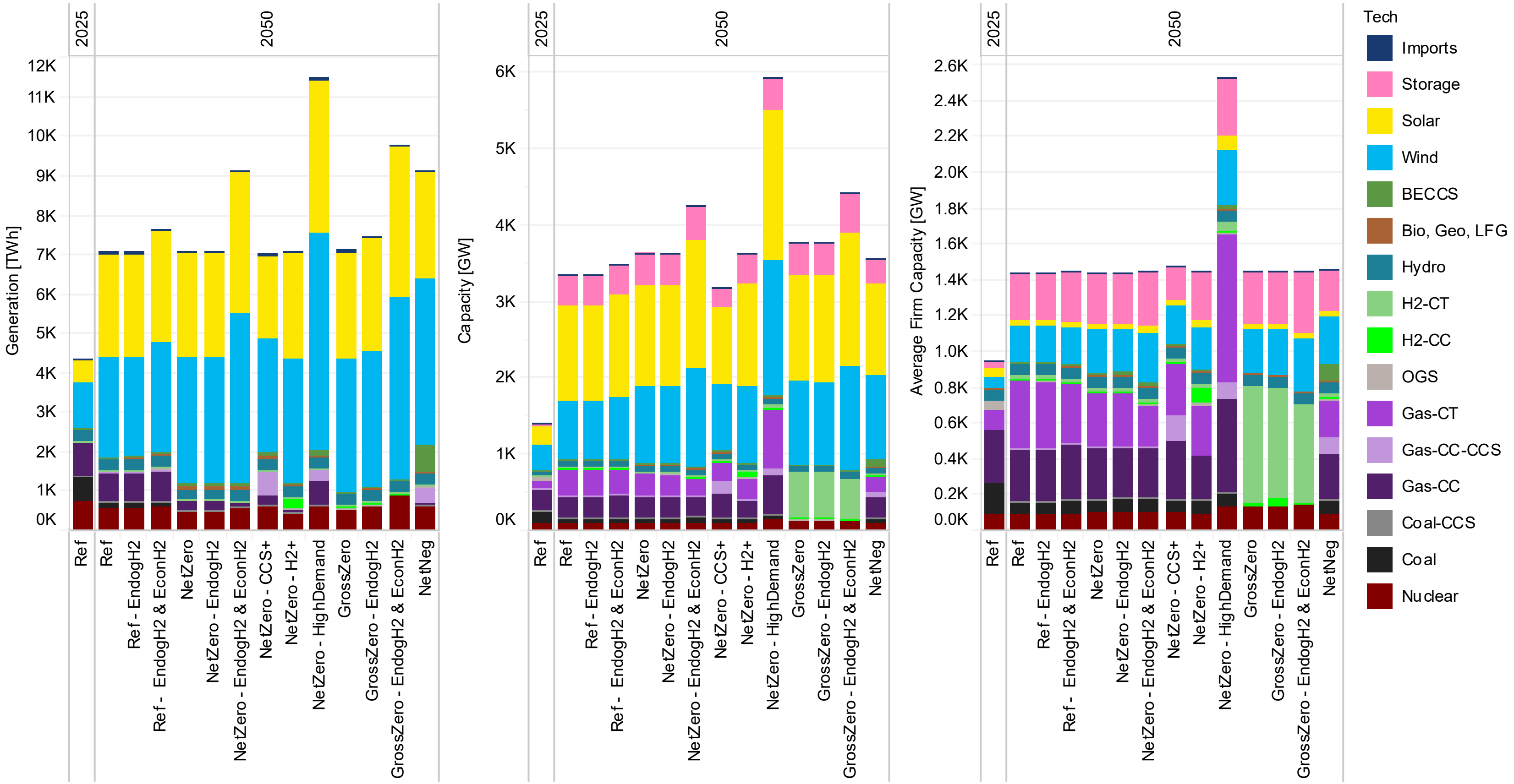

Figure S7. Capacity, generation, and firm capacity in the Reference in 2025 and all cases in 2050. 2025 results are shown only for the Reference case as results are identical across scenarios in 2025. Imports are net imports from Canada. Storge and transmission losses are not shown.

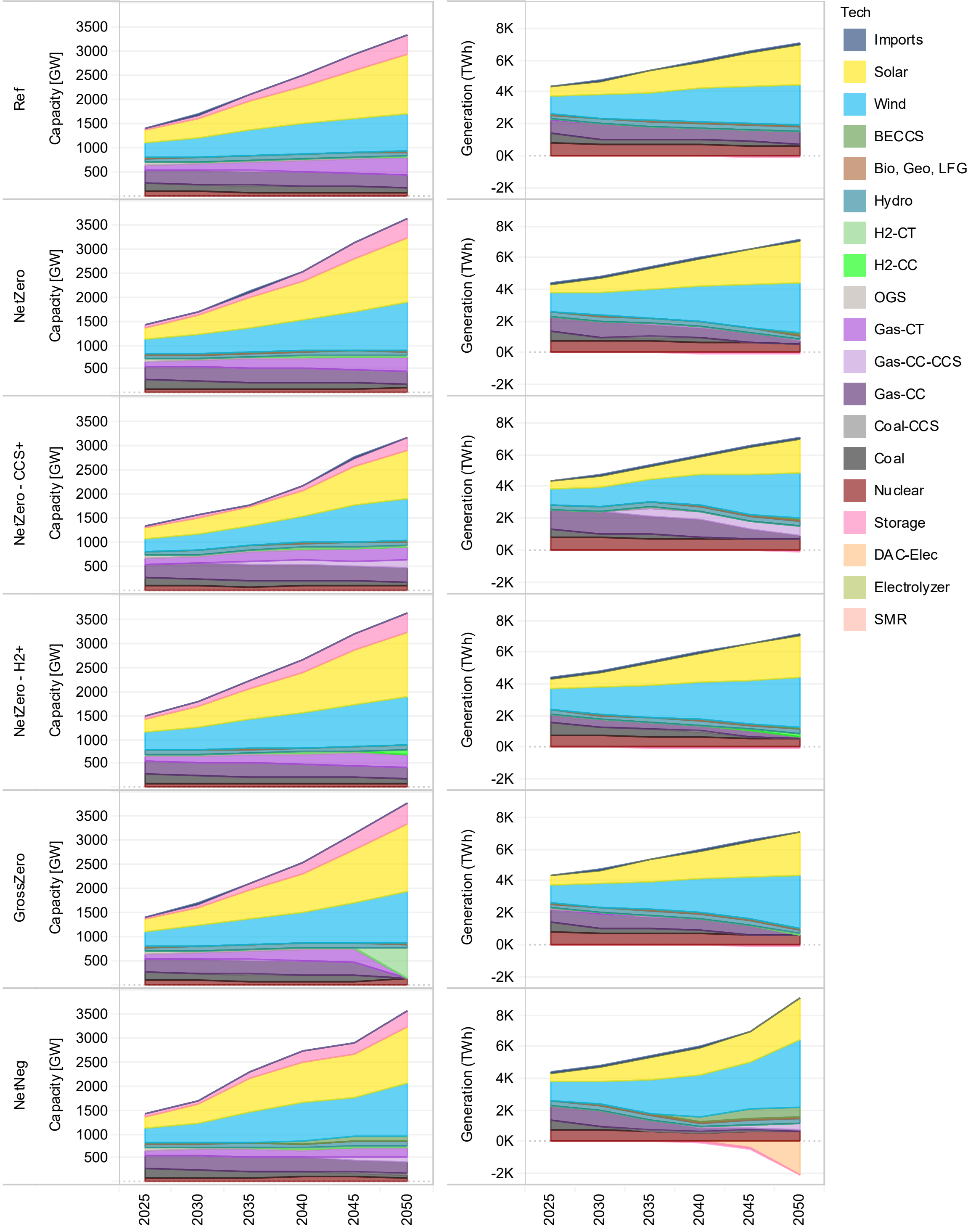

Figure S8. Capacity (left) and generation (right) evolution across all core scenarios

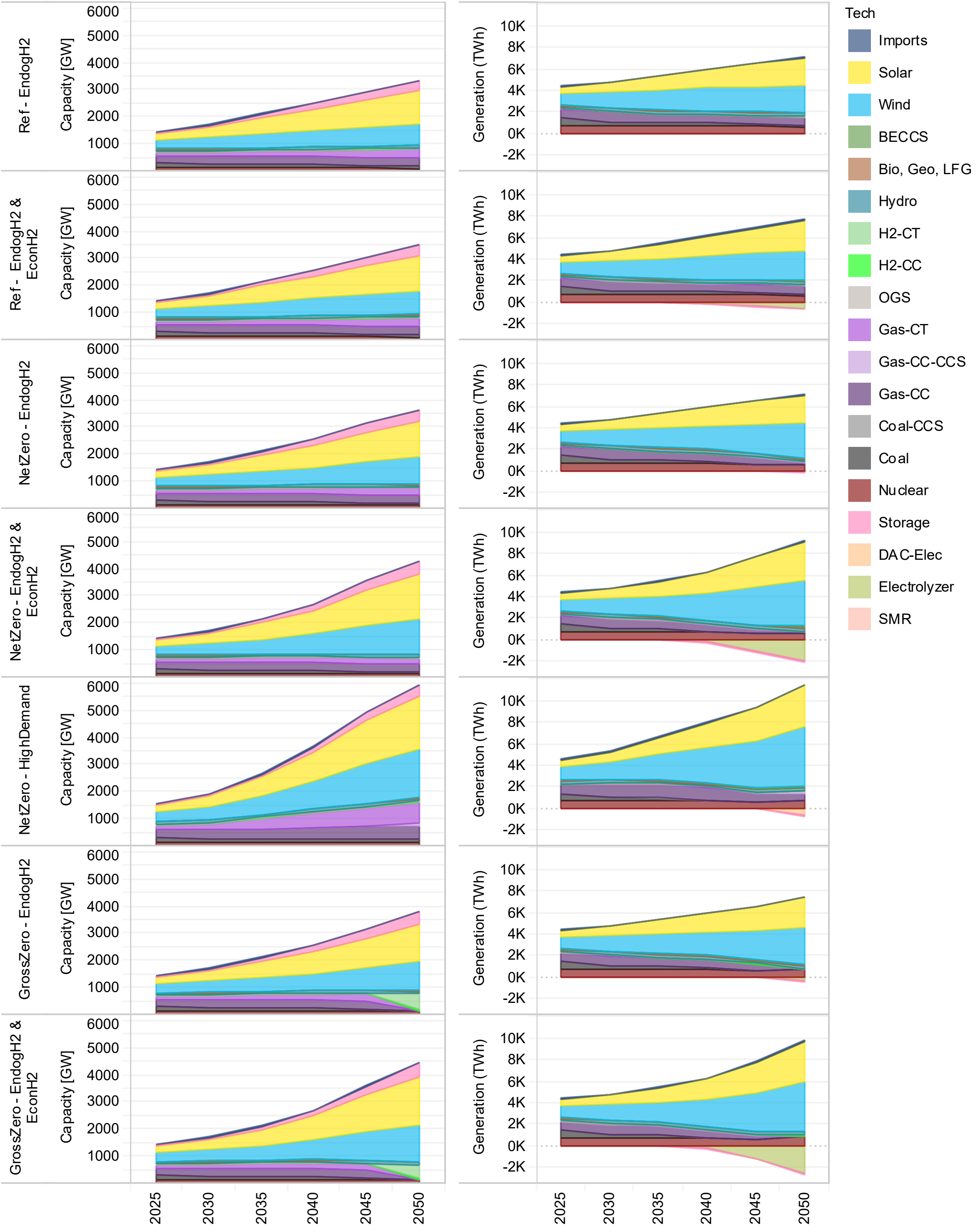

Figure S9. Capacity (left) and generation (right) evolution across all sensitivity scenarios.

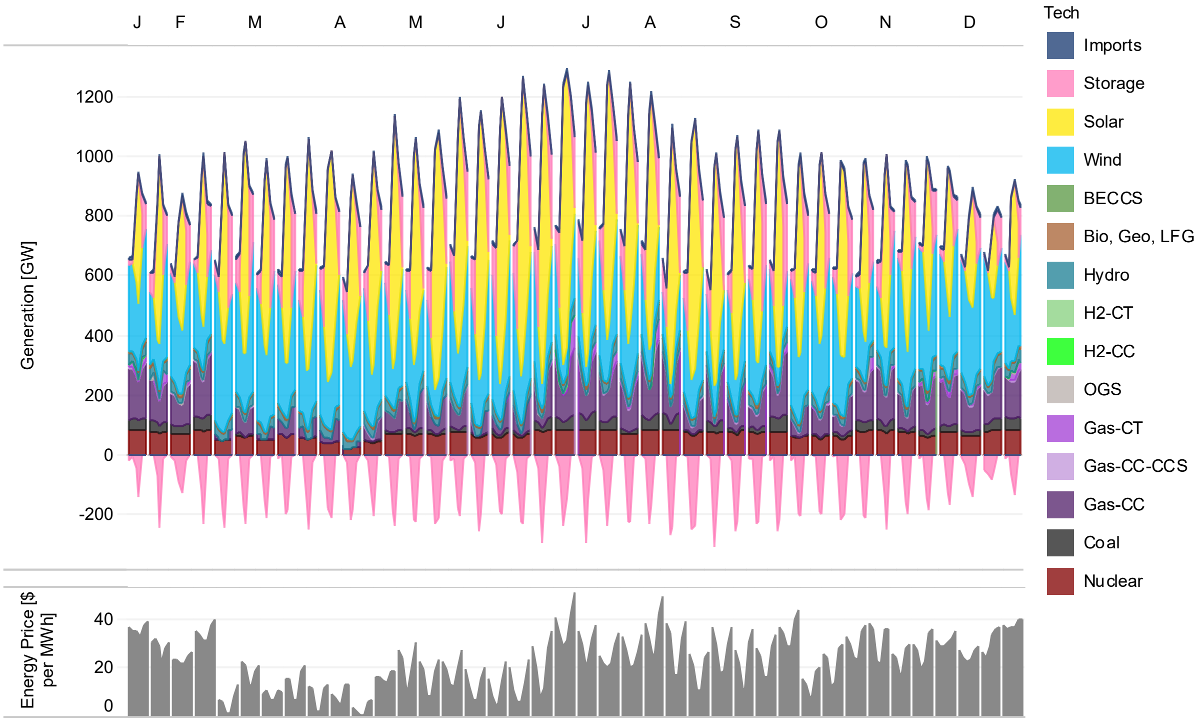

Figure S10. National generation dispatch and national average energy price by timeslice in the Ref scenario..

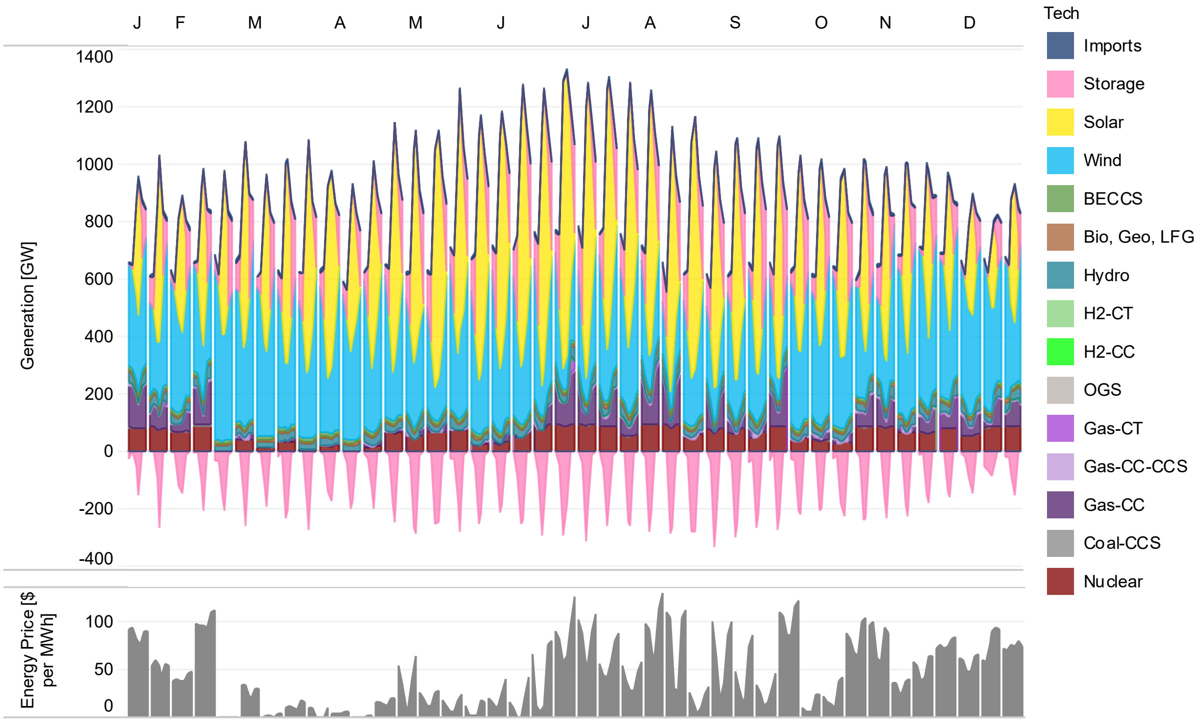

Figure S11. National generation dispatch and national average energy price by time slice in the NetZero scenario.

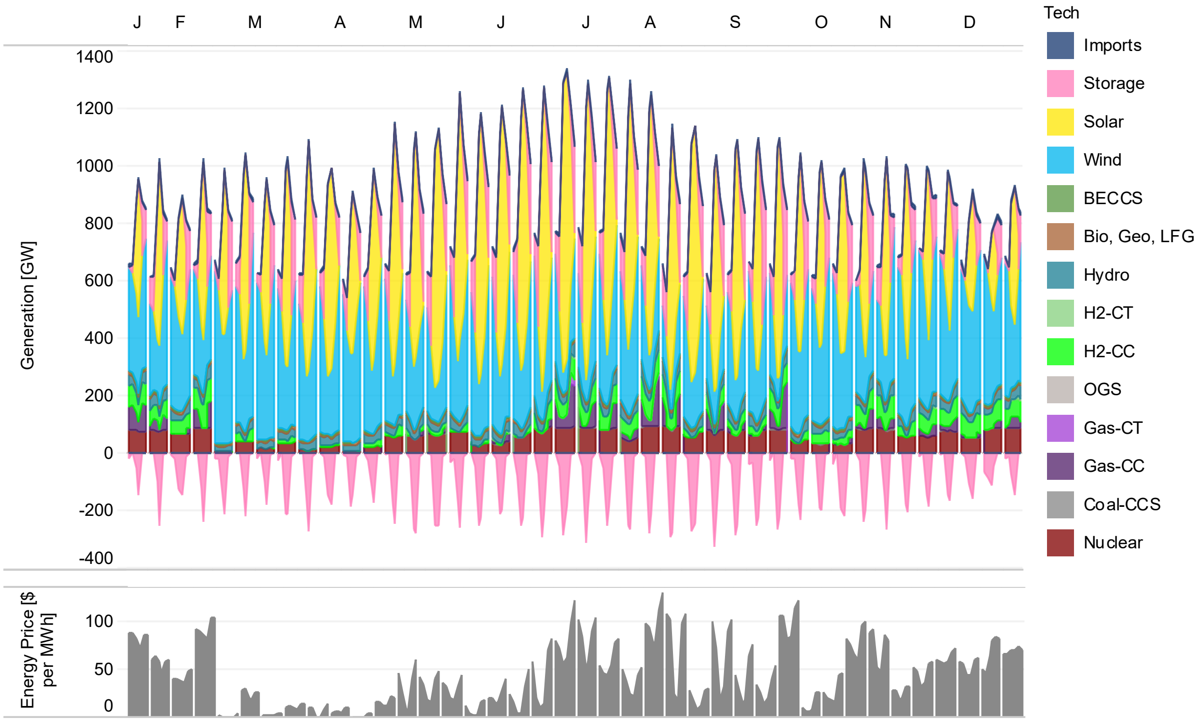

Figure S12. National generation dispatch and national average energy price by time slice in the NetZero-H2+ scenario.

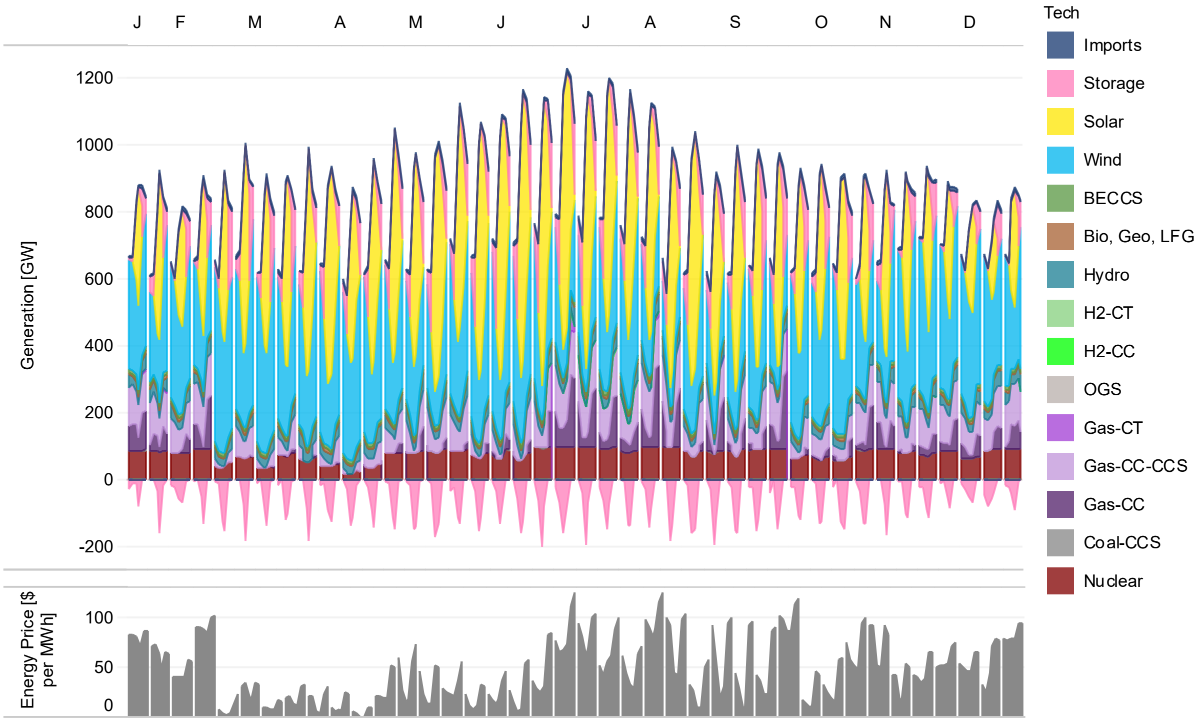

Figure S13. National generation dispatch and national average energy price by time slice in the NetZero-CCS+ scenario.

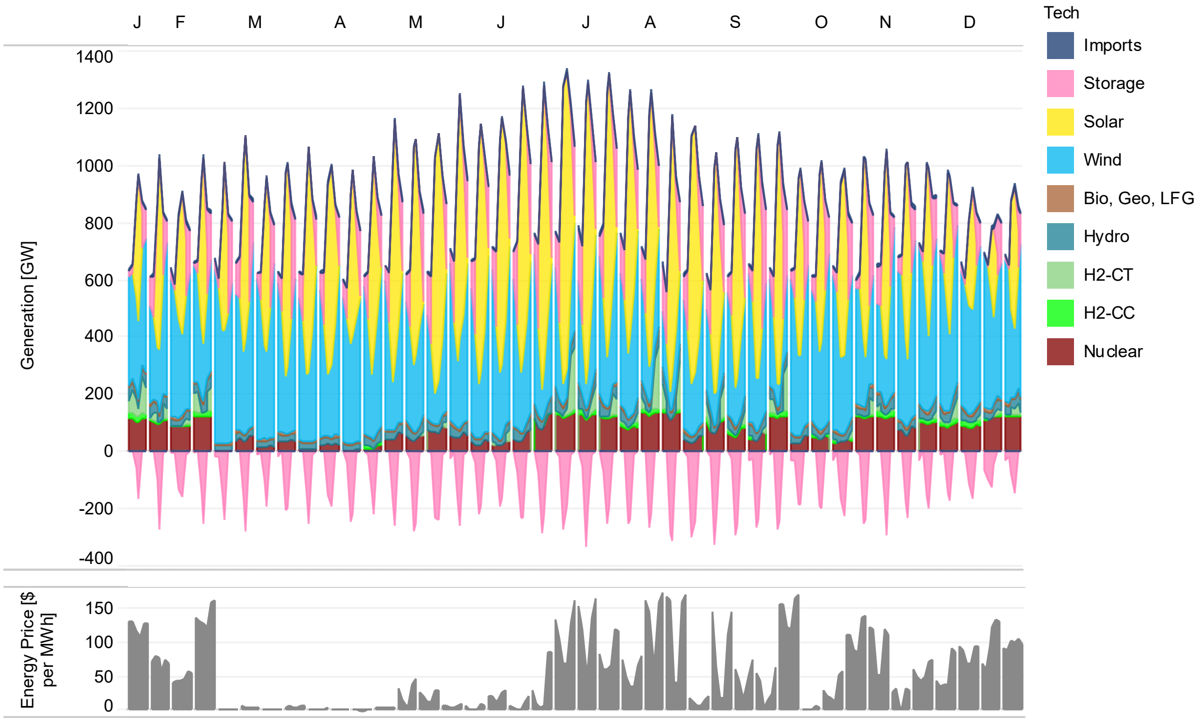

Figure S14. National generation dispatch and national average energy price by time slice in the GrossZero scenario.

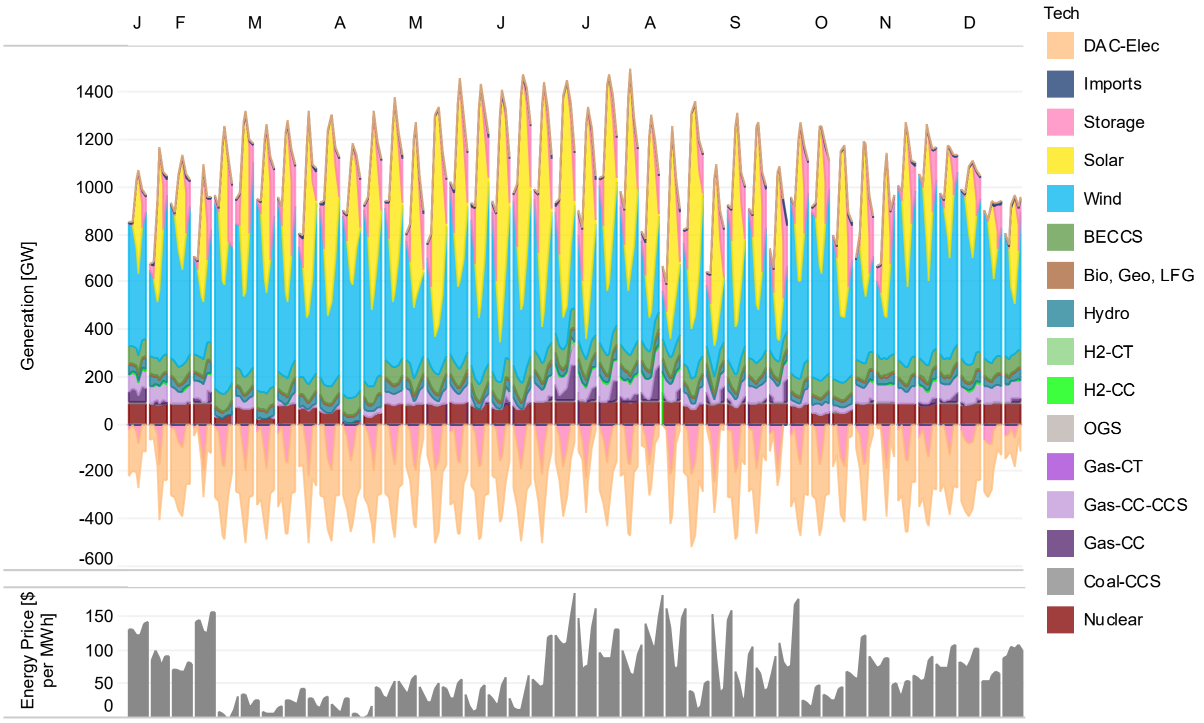

Figure S15. National generation dispatch and national average energy price by time slice in the NetNeg scenario

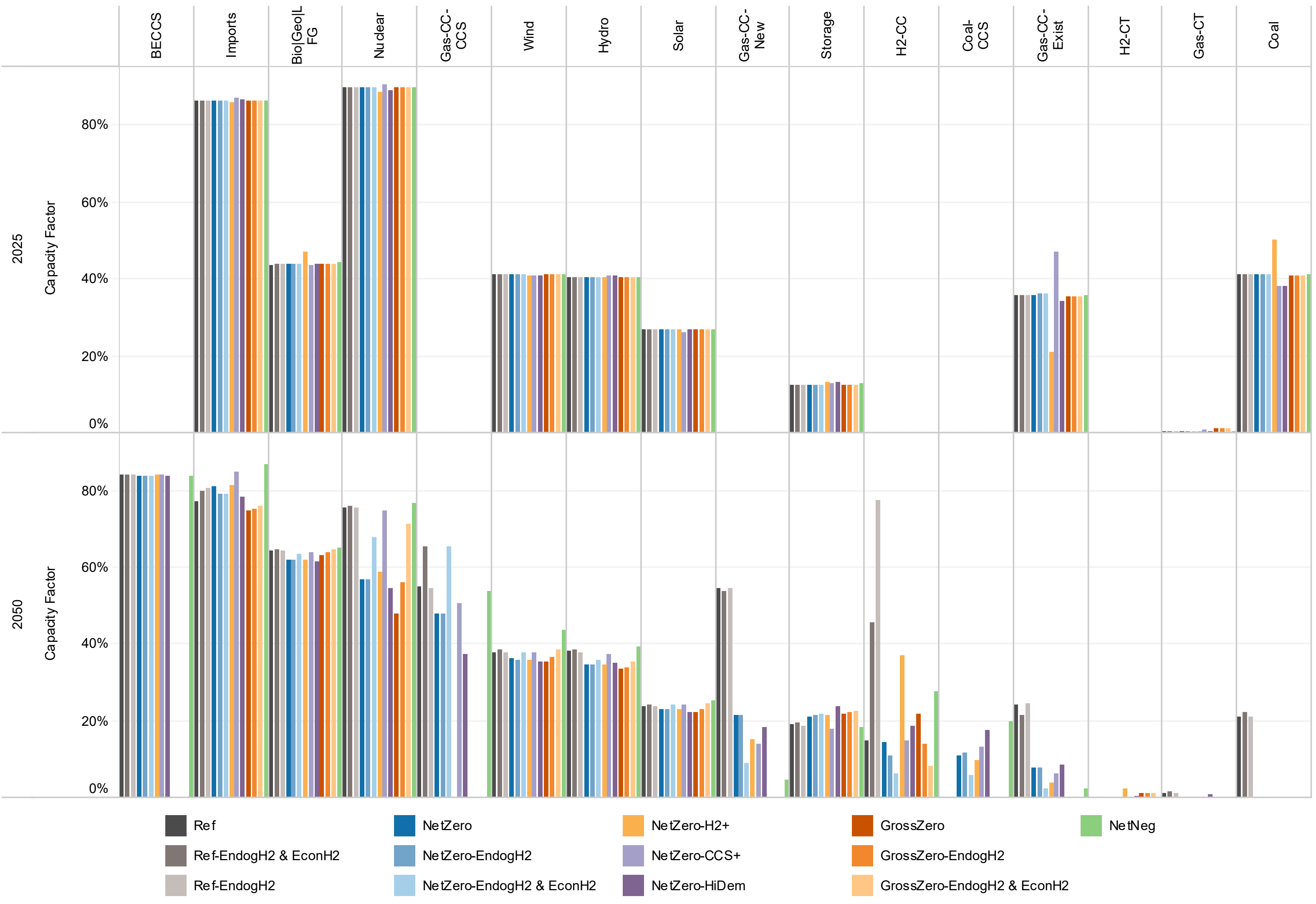

Figure S16. Annual capacity factor by technology type across all scenarios in 2025 (top row) and 2050 (bottom row).

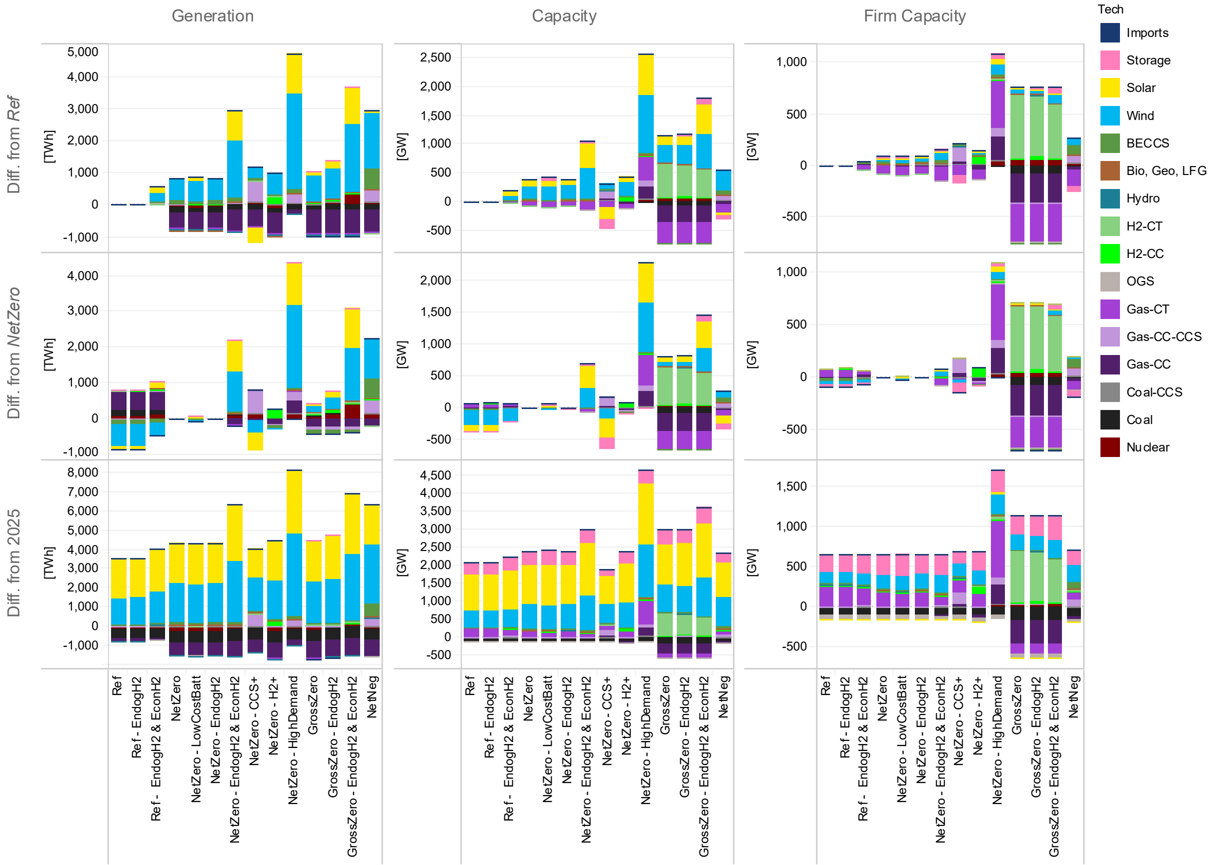

Figure S17. Differences in generation, capacity, and firm capacity in 2050 by scenario relative to the Reference Case (top row), NetZero case (middle row), and from 2025 (bottom row).

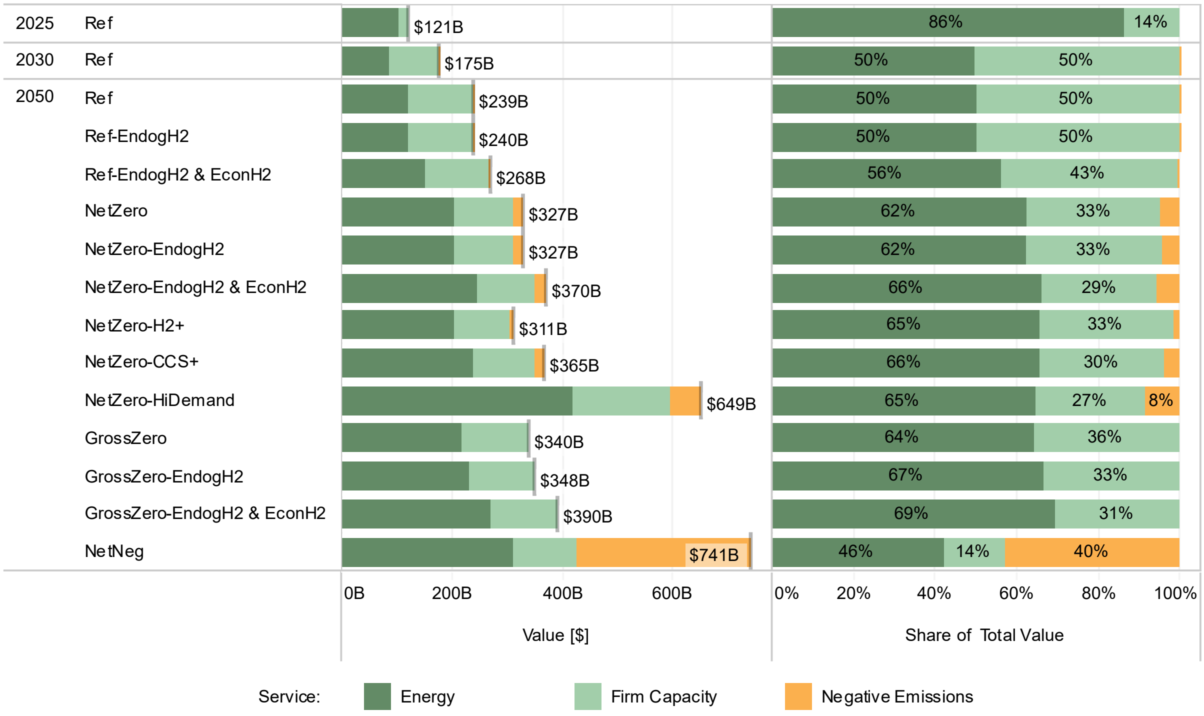

Figure S18. Total value of services and share of total value of services by service type and scenario.

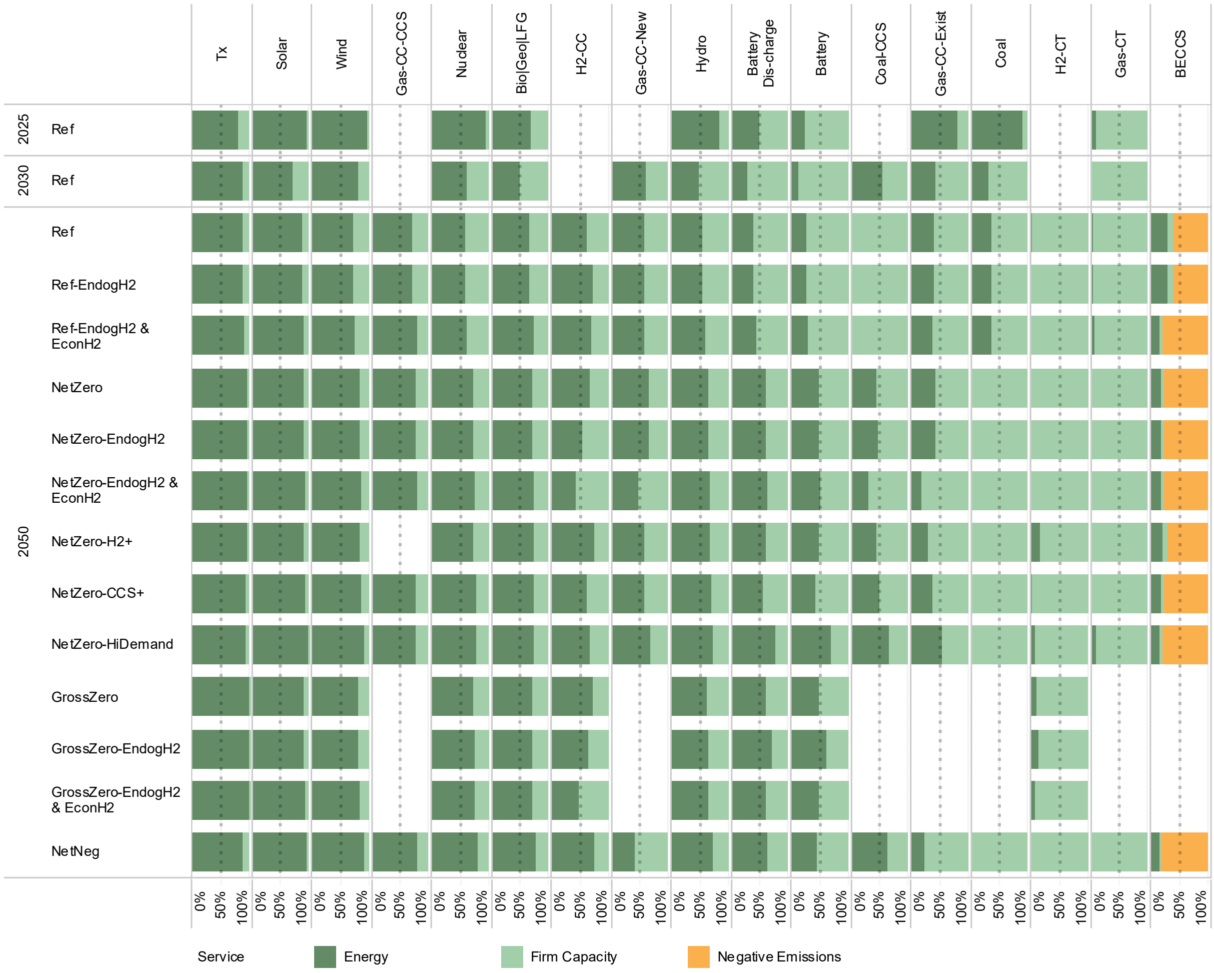

Figure S19. Share of each technology's total system value by service across scenarios in 2050, and for the Reference case in 2025 and 2030. Except for BECCS, technologies are ordered from highest to lowest energy value share under the NetZero case in 2050. For technologies that provide and consume the same service (storage, transmission), net value is shown in this chart, with the gross value for storage shown as a separate column (Battery-Discharge). Gross value of storage is the value associated with discharging (electricity sales) excluding the cost of charging (electricity purchases). The dotted vertical reference lines indicate a 50% share

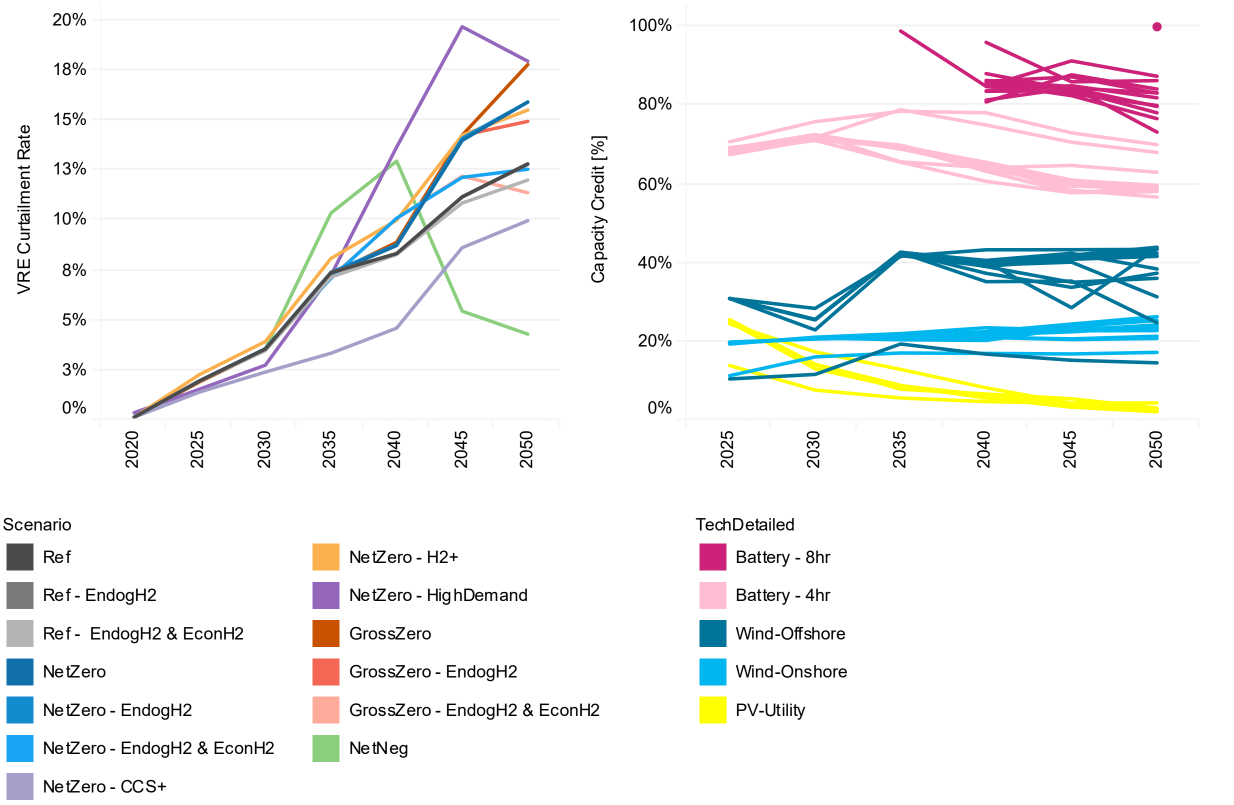

Figure S20. VRE (total wind and solar) curtailment rate by year and scenario (left); average capacity credit of VRE and battery resources by technology type, year, and scenario (right). Lines of identical color in the capacity credit figure indicate values in the six core scenarios..

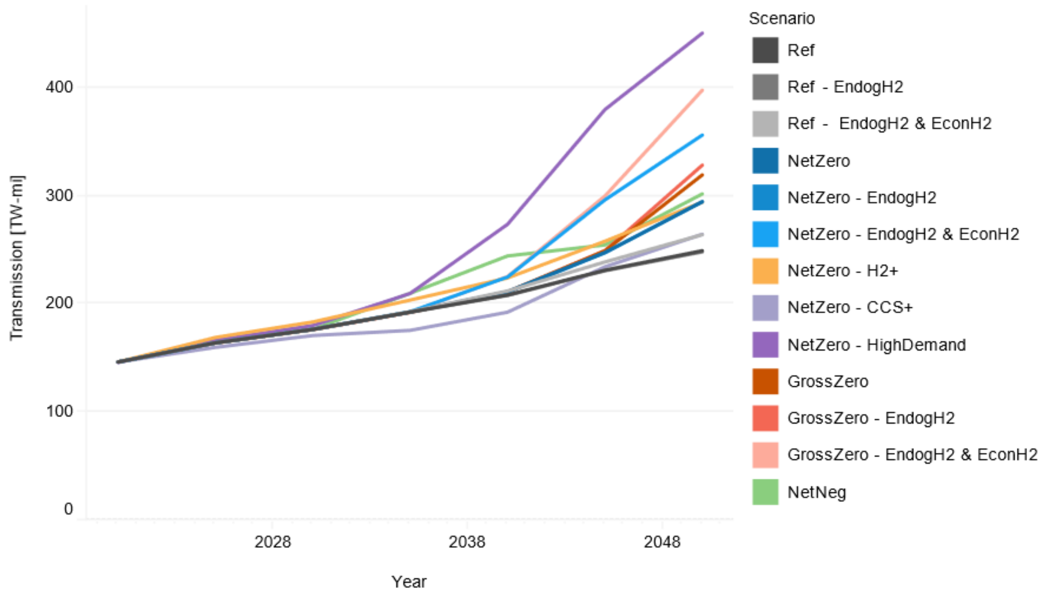

Figure S21. Total transmission capacity by year and scenario.

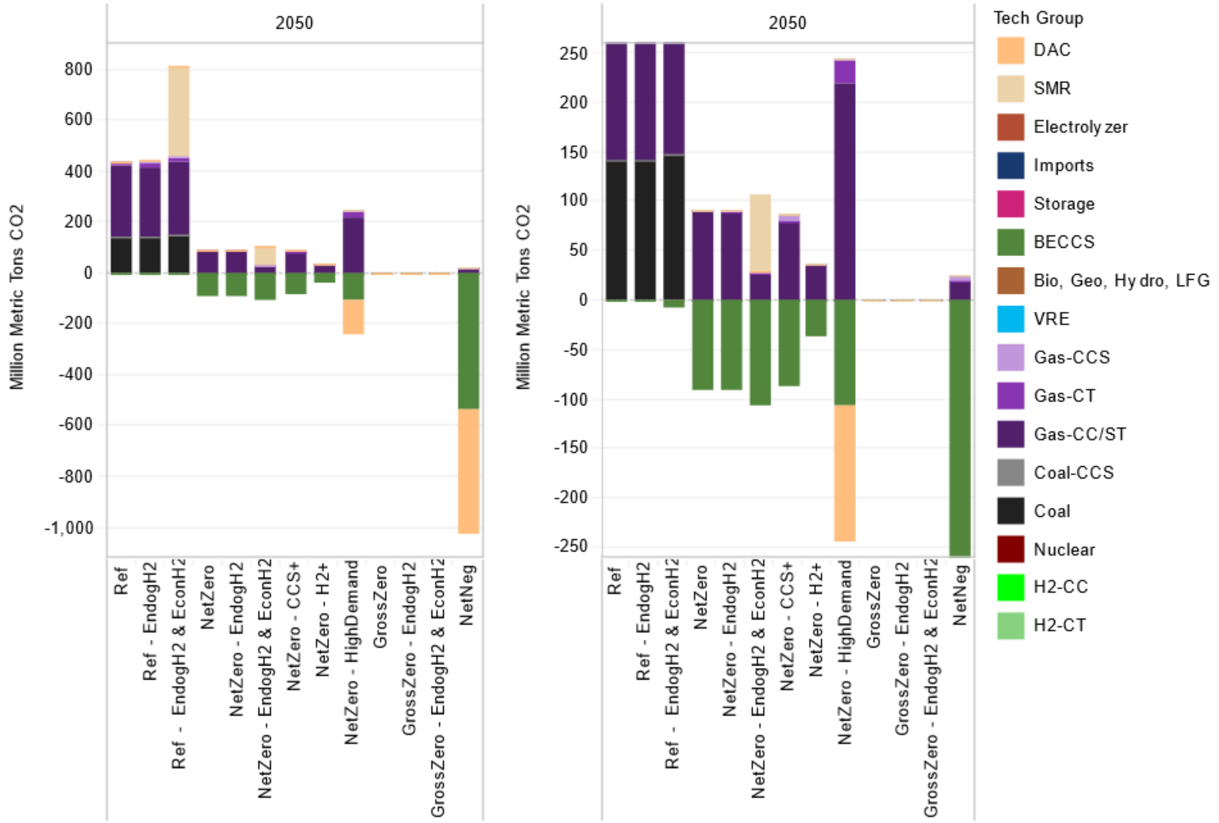

Figure S22. CO_2 emissions in 2050 by scenario and technology. The scale of the Y-axis is reduced in the panel on the right, which leads to truncation in two of the cases.

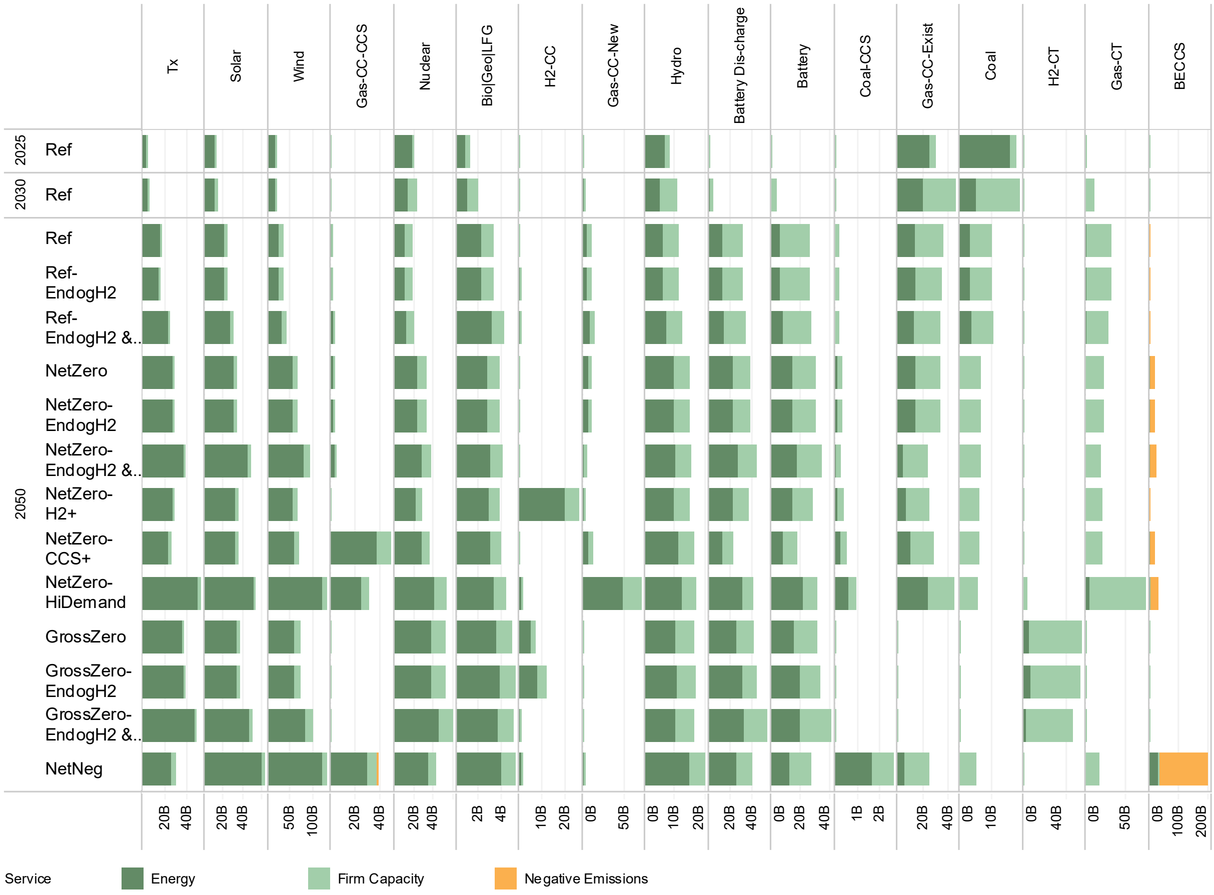

Figure S23. System value (2020$) of each technology by service.

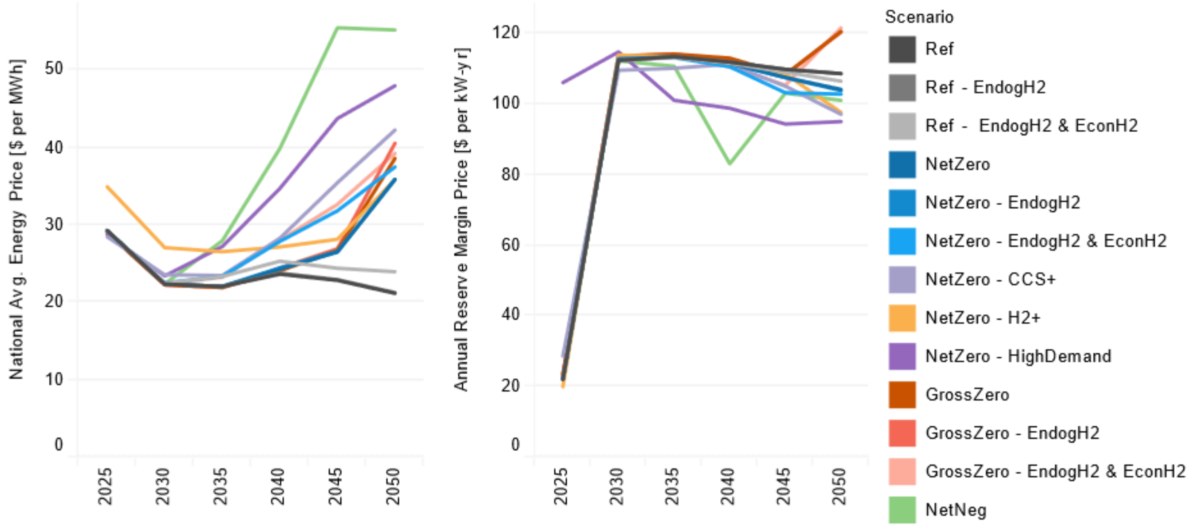

Figure S24. National generation-weighted average wholesale energy price (left) and national average maximum reserve margin (firm capacity) price (right).

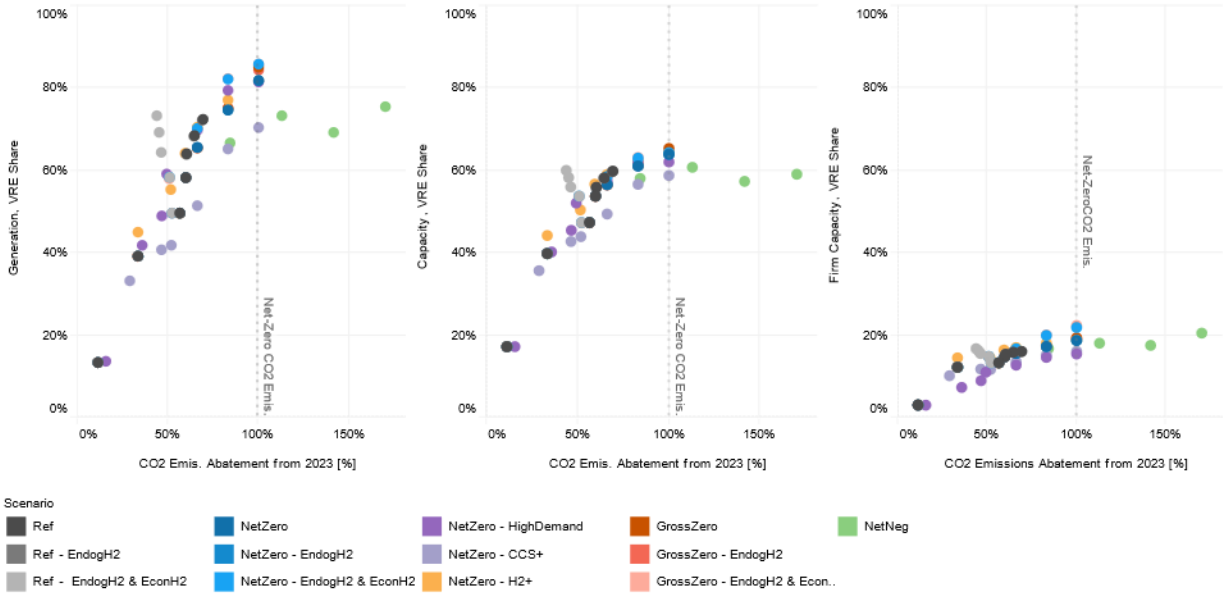

Figure S25. VRE share of generation, capacity, and firm capacity by scenario as a function of CO2 emissions abatement relative to 2023 power sector emissions.
